# Supplementary material for: Diversity of phlebotomine sand flies and molecular detection of trypanosomatids in Brumadinho, Minas Gerais, Brazil
Source: PLoS One. 2020 Jun 24;15(6):e0234445. doi: 10.1371/journal.pone.0234445 (PMC7314019; doi:10.1371/journal.pone.0234445)
Supplement: Attachment — Submitted filename: PONE-D-20-05836_R1_reviewer1.pdf [file pone.0234445.s003.pdf]

PLOS ONE

Diversity of phlebotomine sand flies and molecular detection of trypanosomatids species in Brumadinho, Minas Gerais, Brazil

--Manuscript Draft--

Manuscript Number:	PONE-D-20-05836R1
Article Type:	Research Article
Full Title:	Diversity of phlebotomine sand flies and molecular detection of trypanosomatids species in Brumadinho, Minas Gerais, Brazil
Short Title:	Phlebotomine sand flies and trypanosomatids in area of leishmaniases in Brazil
Corresponding Author:	José Dilermando Andrade Filho<br>Centro de Pesquisas René Rachou/Fundação Oswaldo Cruz<br>Belo Horizonte, Minas Gerais BRAZIL
Keywords:	Leishmaniases; Leishmania braziliensis; Leishmania amazonensis; Lutzomyia longipalpis; Nyssomyia whitmani; Trypanosomatidae
Abstract:	This study aimed to investigate the phlebotomine sand fly fauna and perform the molecular detection of trypanosomatids in sand flies collected in the Casa Branca district of Brumadinho municipality, located in the metropolitan region of Belo Horizonte, state of Minas Gerais, Brazil, an endemic area of visceral (VL) and tegumentary leishmaniases (TL). Samples were collected bimonthly from May 2013 to July 2014 using two HP light traps exposed in nine peridomiciliary areas with previous reports of VL or TL (total 18 traps) for three consecutive nights. DNA was extracted from female sand flies and subjected to ITS1-PCR. Subsequently DNA sequencing was conducted on PCR products for species level identification. A total of 23 species were found among 16,771 sand flies collected. The most abundant species was Nyssomyia whitmani (Antunes & Coutinho, 1939) (70.9%), followed by Lutzomyia longipalpis (Lutz & Neiva, 1912) (15.2%) and Migonemyia migonei (França, 1920) (9.1%). A total of 4,913 females were analyzed individually or in pools of 1-20 specimens for the molecular detection of Leishmania parasites. The DNA of Leishmania amazonensis was detected in four pools, belonging to Nyssomyia whitmani ; DNA of Le . braziliensis was detected in one pool of Psychodopygus lloydi , and Leishmania sp. was detected pools of Nyssomyia whitmani (7) and Lutzomyia longipalpis (1) In addition, the DNA of Herpetomonas sp. and Crithidia sp . was detected in female sand flies and identified using ITS1-PCR. Our results suggest that Ny . whitmani is a vector in the study area. In addition, the molecular detection of Le . amazonensis suggests the presence of this species in a sylvatic cycle between vertebrate and invertebrate hosts in the region of Casa Branca. Our data also reveal the occurrence of other non- Leishmania trypanosomatids in sand flies in Casa Branca District.
Order of Authors:	Aline Tanure
	Felipe Dutra Rêgo
	Gabriel Barbosa Tonelli
	Aldenise Martins Campos
	Paloma Helena Fernandes Shimabukuro
	Célia Maria Ferreira Gontijo
	Gustavo Fontes Paz
	José Dilermando Andrade Filho
Response to Reviewers:	PONE-D-20-05836<br>Diversity of phlebotomine sand flies and molecular detection of trypanosomatids species in Brumadinho, Minas Gerais, Brazil<br>PLOS ONE

Powered by Editorial Manager® and ProduXion Manager® from Aries Systems Corporation

Dear Vyacheslav Yurchenko,

Below are our answers to requests from the Editor and the PLOS ONE technical team.

Thanks

Additional Editor Comments (if provided):

Reading the manuscript of Tanure at al I noticed the huge unbalance in the literature citations. Authors (almost exclusively) cite Brazilian papers effectively ignoring the huge amount of work on this topic, which has been done in other parts of the world. I invite authors to correct this discrepancy and submit a revised version for peer review.
V, Yurchenko
The Editor is right, there were many references in Portuguese that could be replaced by others, thanks for the suggestion.

Journal Requirements:
When submitting your revision, we need you to address these additional requirements:
1. Please ensure that your manuscript meets PLOS ONE's style requirements, including those for file naming. The PLOS ONE style templates can be found at http://www.plosone.org/attachments/PLOSOne_formatting_sample_main_body.pdf and http://www.plosone.org/attachments/PLOSOne_formatting_sample_title_authors_affiliations.pdf
We understand that our article and files are based on the PLOS ONE style.

2. One of the noted authors is a group or consortium [Grupo de Estudos em Leishmanioses]. In addition to naming the author group, please list the individual authors and affiliations within this group in the acknowledgments section of your manuscript. Please also indicate clearly a lead author for this group along with a contact email address.
Grupo de Estudos em Leishmanioses is the author's affiliation, a name similar to "laboratory". All authors belong to the leishmaniasis study group (Grupo de Estudos em Leishmanioses)

3. Please amend your list of authors on the manuscript to ensure that each author is linked to an affiliation. Authors' affiliations should reflect the institution where the work was done (if authors moved subsequently, you can also list the new affiliation stating "current affiliation:…." as necessary).
All authors are listed and with their affiliation
4. Please ensure that you refer to Figure 2 in your text as, if accepted, production will need this reference to link the reader to the figure.
We have included the reference to figure 2 in the text, line 214
5. We note that Figure 1 in your submission contain map images which may be copyrighted. All PLOS content is published under the Creative Commons Attribution License (CC BY 4.0), which means that the manuscript, images, and Supporting Information files will be freely available online, and any third party is permitted to access, download, copy, distribute, and use these materials in any way, even commercially, with proper attribution. For these reasons, we cannot publish previously copyrighted maps or satellite images created using proprietary data, such as Google software (Google Maps, Street View, and Earth). For more information, see our copyright guidelines: http://journals.plos.org/plosone/s/licenses-and-copyright.
We require you to either (1) present written permission from the copyright holder to publish these figures specifically under the CC BY 4.0 license, or (2) remove the figures from your submission:
1. You may seek permission from the original copyright holder of Figure 1 to publish the content specifically under the CC BY 4.0 license.
We recommend that you contact the original copyright holder with the Content Permission Form (http://journals.plos.org/plosone/s/file?id=7c09/content-permission-form.pdf) and the following text:
"I request permission for the open-access journal PLOS ONE to publish XXX under the Creative Commons Attribution License (CCAL) CC BY 4.0 (http://creativecommons.org/licenses/by/4.0/). Please be aware that this license allows

	unrestricted use and distribution, even commercially, by third parties. Please reply and provide explicit written permission to publish XXX under a CC BY license and complete the attached form.”<br>Please upload the completed Content Permission Form or other proof of granted permissions as an "Other" file with your submission.<br>In the figure caption of the copyrighted figure, please include the following text: “Reprinted from [ref] under a CC BY license, with permission from [name of publisher], original copyright [original copyright year].”<br>2. If you are unable to obtain permission from the original copyright holder to publish these figures under the CC BY 4.0 license or if the copyright holder’s requirements are incompatible with the CC BY 4.0 license, please either i) remove the figure or ii) supply a replacement figure that complies with the CC BY 4.0 license. Please check copyright information on all replacement figures and update the figure caption with source information. If applicable, please specify in the figure caption text when a figure is similar but not identical to the original image and is therefore for illustrative purposes only.<br>We will provide copyright permission for figure 1 and include it in the submission system<br><br>Thank you!!!!<br><br>José Dilermando Andrade Filho
**Additional Information:**	
**Question**	**Response**
**Financial Disclosure**<br><br>Enter a financial disclosure statement that describes the sources of funding for the work included in this submission. Review the submission guidelines for detailed requirements. View published research articles from *PLOS ONE* for specific examples.<br><br>This statement is required for submission and **will appear in the published article** if the submission is accepted. Please make sure it is accurate.	JDAF - 302701/2016-8 Conselho Nacional de Desenvolvimento Científico e Tecnológico (www.cnpq.br)<br>JDAF - PPM-00792-18 Fundação de Amparo à Pesquisa de Minas Gerais (www.fapemig.br)<br>AT - Finance Code 001 Coordenação de Aperfeiçoamento de Pessoal de Nível Superior (www.capes.gov.br)<br><br>The funders had no role in study design, data collection and analysis, decision to publish, or preparation of the manuscript.

**Unfunded studies**<br>Enter: *The author(s) received no specific funding for this work.*<br><br>**Funded studies**<br>Enter a statement with the following details:<br>• Initials of the authors who received each award<br>• Grant numbers awarded to each author<br>• The full name of each funder<br>• URL of each funder website<br>• Did the sponsors or funders play any role in the study design, data collection and analysis, decision to publish, or preparation of the manuscript?<br>• **NO** - Include this sentence at the end of your statement: *The funders had no role in study design, data collection and analysis, decision to publish, or preparation of the manuscript.*<br>• **YES** - Specify the role(s) played.<br><br>* typeset	
**Competing Interests**<br><br>Use the instructions below to enter a competing interest statement for this submission. On behalf of all authors, disclose any competing interests that could be perceived to bias this work—acknowledging all financial support and any other relevant financial or non-financial competing interests.<br><br>This statement **will appear in the published article** if the submission is accepted. Please make sure it is accurate. View published research articles from *PLOS ONE* for specific examples.	The authors have declared that no competing interests exist.

Powered by Editorial Manager® and ProduXion Manager® from Aries Systems Corporation

**NO authors have competing interests**<br><br>Enter: *The authors have declared that no competing interests exist.*<br><br>**Authors with competing interests**<br><br>Enter competing interest details beginning with this statement:<br><br>*I have read the journal's policy and the authors of this manuscript have the following competing interests: [insert competing interests here]*<br><br>* typeset	
**Ethics Statement**<br><br>Enter an ethics statement for this submission. This statement is required if the study involved:<br><br>• Human participants<br>• Human specimens or tissue<br>• Vertebrate animals or cephalopods<br>• Vertebrate embryos or tissues<br>• Field research<br><br>Write "N/A" if the submission does not require an ethics statement.<br><br>General guidance is provided below. Consult the submission guidelines for detailed instructions. **Make sure that all information entered here is included in the Methods section of the manuscript.**	The owners of the residences where the sand flies were collected authorized verbally the installation of the traps during the period of study.<br>The collections were carried out with the due permission of the Brazilian environmental agencies (SISBIO number 15237-2). The sand flies were deposited in the Coleção de Flebotomíneos of the Instituto René Rachou/Fiocruz (FIOCRUZ/COLFLEB).

Powered by Editorial Manager® and ProduXion Manager® from Aries Systems Corporation

**Format for specific study types**<br><br>**Human Subject Research (involving human participants and/or tissue)**<br>• Give the name of the institutional review board or ethics committee that approved the study<br>• Include the approval number and/or a statement indicating approval of this research<br>• Indicate the form of consent obtained (written/oral) or the reason that consent was not obtained (e.g. the data were analyzed anonymously)<br><br>**Animal Research (involving vertebrate animals, embryos or tissues)**<br>• Provide the name of the Institutional Animal Care and Use Committee (IACUC) or other relevant ethics board that reviewed the study protocol, and indicate whether they approved this research or granted a formal waiver of ethical approval<br>• Include an approval number if one was obtained<br>• If the study involved *non-human primates*, add *additional details* about animal welfare and steps taken to ameliorate suffering<br>• If anesthesia, euthanasia, or any kind of animal sacrifice is part of the study, include briefly which substances and/or methods were applied<br><br>**Field Research**<br><br>Include the following details if this study involves the collection of plant, animal, or other materials from a natural setting:<br>• Field permit number<br>• Name of the institution or relevant body that granted permission	
**Data Availability**<br><br>Authors are required to make all data underlying the findings described fully available, without restriction, and from the time of publication. PLOS allows rare exceptions to address legal and ethical concerns. See the PLOS Data Policy and FAQ for detailed information.	Yes - all data are fully available without restriction

A Data Availability Statement describing where the data can be found is required at submission. Your answers to this question constitute the Data Availability Statement and **will be published in the article**, if accepted.<br><br>**Important:** Stating 'data available on request from the author' is not sufficient. If your data are only available upon request, select 'No' for the first question and explain your exceptional situation in the text box.<br><br>Do the authors confirm that all data underlying the findings described in their manuscript are fully available without restriction?	
**Describe where the data may be found in full sentences. If you are copying our sample text, replace any instances of XXX with the appropriate details.**<br><br>• If the data are **held or will be held in a public repository**, include URLs, accession numbers or DOIs. If this information will only be available after acceptance, indicate this by ticking the box below. For example: *All XXX files are available from the XXX database (accession number(s) XXX, XXX.*).<br>• If the data are all contained **within the manuscript and/or Supporting Information files**, enter the following: *All relevant data are within the manuscript and its Supporting Information files.*<br>• If neither of these applies but you are able to provide **details of access elsewhere**, with or without limitations, please do so. For example:<br><br>*Data cannot be shared publicly because of [XXX]. Data are available from the XXX Institutional Data Access / Ethics Committee (contact via XXX) for researchers who meet the criteria for access to confidential data.*<br><br>*The data underlying the results presented in the study are available from (include the name of the third party*	All relevant data are within the manuscript and its Supporting Information files

Powered by Editorial Manager® and ProduXion Manager® from Aries Systems Corporation

and contact information or URL).<br>• This text is appropriate if the data are owned by a third party and authors do not have permission to share the data.<br><br>* typeset	
Additional data availability information:	Tick here if the URLs/accession numbers/DOIs will be available only after acceptance of the manuscript for publication so that we can ensure their inclusion before publication.

Powered by Editorial Manager® and ProduXion Manager® from Aries Systems Corporation

Manuscript Click here to access/download;Manuscript;Manuscript1.doc

Short title: Phlebotomine sand flies and trypanosomatids in area of leishmaniases in Brazil

Diversity of phlebotomine sand flies and molecular detection of trypanosomatids species in Brumadinho, Minas Gerais, Brazil

Aline Tanure, Felipe Dutra Rêgo, Gabriel Barbosa Tonelli, Aldenise Martins Campos, Paloma Helena Fernandes Shimabukuro, Célia Maria Ferreira Gontijo, Gustavo Fontes Paz, José Dilermando Andrade-Filho*

Grupo de Estudos em Leishmanioses – Instituto René Rachou – FIOCRUZ Minas – Av. Augusto de Lima, 1715 Barro Preto, CEP 30190–002, Belo Horizonte, Minas Gerais, Brasil

*corresponding author

E-mail: jose.andrade@fiocruz.br

Abstract

This study aimed to investigate the phlebotomine sand fly fauna and perform the molecular detection of trypanosomatids in sand flies collected in the Casa Branca district of Brumadinho municipality, located in the metropolitan region of Belo Horizonte, state of Minas Gerais, Brazil, an endemic area of visceral (VL) and tegumentary leishmaniases (TL). Samples were collected bimonthly from May 2013 to July 2014 using two HP light traps exposed in nine peridomiciliary areas with previous reports of VL or TL (total 18 traps) for three consecutive nights. DNA was extracted from female sand flies and subjected to ITS1-PCR. Subsequently DNA sequencing was conducted on PCR products for species level identification. A total of 23 species were found among 16,771 sand flies collected. The most abundant species was *Nyssomyia whitmani* (Antunes & Coutinho, 1939) (70.9%), followed by *Lutzomyia longipalpis* (Lutz & Neiva, 1912) (15.2%) and *Migonemyia migonei* (França, 1920) (9.1%). A total of 4,913 females were analyzed individually or in pools of 1-20 specimens for the molecular detection of *Leishmania* parasites. The DNA of *Leishmania amazonensis* was detected in four pools, belonging to *Nyssomyia whitmani*; DNA of *Le. braziliensis* was detected in one pool of *Psychodopygus lloydi*, and *Leishmania* sp. was detected pools of *Nyssomyia whitmani* (7) and *Lutzomyia longipalpis* (1) In addition, the DNA of *Herpetomonas* sp. and *Crithidia* sp. was detected in female sand flies and identified using ITS1-PCR. Our results suggest that *Ny. whitmani* is a vector in the study area. In addition, the molecular detection of *Le. amazonensis* suggests the presence of this species in a sylvatic cycle between vertebrate and invertebrate hosts in the region of Casa Branca. Our data also reveal the occurrence of other non-*Leishmania* trypanosomatids in sand flies in Casa Branca District.

Key words: Leishmaniases; *Leishmania braziliensis*; *Leishmania amazonensis*; *Lutzomyia longipalpis*; *Nyssomyia whitmani*; Trypanosomatidae

Introduction

Phlebotomine sand flies (Diptera: Psychodidae: Phlebotominae) are known to be natural hosts of etiological agents such as bacteria, viruses and protozoa [1, 2]. Among these agents, are species of the genus *Leishmania*, which are transmitted during blood feeding by female sand flies [3]. In the New World, some genera of sand flies (*Lutzomyia*, *Psychodopygus*, *Pintomyia*, *Nyssomyia*, among others) are involved in the transmission of these parasites to vertebrate hosts [4].

More than 1,000 species of phlebotomine sand flies are known throughout the world, of which about of 530 species are in the Neotropical Region and Brazil has a greater diversity of species of phlebotomine sand flies than any other nation in the world, with approximately 274 species recorded [5].

In Brazil, some species of sand flies are adapted to urban and peri-urban environments, and exhibit a high degree of anthropophily. The species *Lutzomyia longipalpis* is incriminated as the main vector of *Leishmania infantum*, the etiologic agent of visceral leishmaniasis (VL) [6, 7]. Other species of phlebotomine sand flies are important in the transmission of *Leishmania braziliensis*, which causes tegumentary leishmaniasis (TL), such as *Nyssomyia whitmani* and *Nyssomyia intermedia* in the southeast region of Brazil [8, 9].

Other trypanosomatids such as *Endotrypanum*, *Herpetomonas*, *Crithidia*, *Blastocrithidia*, *Leptomonas*, and *Sauroleishmania* are likely to be found in the digestive tract of sand flies, since they may be share the same habitat [10, 11]. Several authors have reported the presence of non-*Leishmania* trypanosomatids DNA in sand

flies by means of molecular techniques [12, 13, 14, 15]. However, the identification of these protozoa has not been considered relevant from the epidemiological point of view. In a recent phylogenetic study, *Leishmania colombiensis*, an important species associated with human cases of TL in South America, was classified in the genus *Endotrypanum* suggesting that the non-*Leishmania* trypanosomatids may also cause leishmaniases and therefore, studies are needed to better explain the role of the sand flies in the transmission of these parasites to humans [16].

Intense urbanization over the last decades has caused changes in natural habitats, especially those occupied by phlebotomine sand flies. Some species are able to withstand several environmental modifications that may occur in natural breeding sites and successfully adapt to urban environments [17, 18]. In some regions, the presence of vector species in the human environment may be associated with the emergence of autochthonous cases of leishmaniases, as occurs in the state of Minas Gerais, where cases have been recorded in several regions [19, 20, 21, 22] including the metropolitan region of Belo Horizonte, Minas Gerais state capital [23, 24]. In this context, the municipality of Brumadinho (Minas Gerais) has reported an increasing number of cases of leishmaniases over the last years. In the period of 2007 to 2014, 28 cases of TL and 13 cases of VL were recorded in the municipality. The district of Casa Branca, which belongs to the municipality of Brumadinho, is responsible for many of these cases, including three TL and four VL cases (Source: Municipal Secretary of Health of Brumadinho, MG). In this area, Pereira et al. [25] recorded the presence of *Leishmania* spp. in rodents and marsupials. Given this situation, this study aimed to describe the sand fly fauna, detect and identify by molecular assay the presence of trypanosomatids in female sand flies from Casa Branca.

Material and Methods

Study area

The municipality of Brumadinho (20º08'36" S and 44º11'59" W) is located within the metropolitan region of Belo Horizonte, approximately 50 km from the state capital. It covers an area of 639,434 km² with a total population of 38,373 inhabitants [26]. The municipality is located in the humid subtropical zone with dry winter and hot summer (Cwa – Köppen's climate classification) situated in an area that includes the "Quadrilátero Ferrífero", an area rich in ferruginous fields, with industrial mining as the primary economic activity.

The study was conducted in the district of Casa Branca (20°6'2.58"S and 44°2'59.45" W), located in the northern part of the municipality of Brumadinho (Figure 1). Casa Branca is also bordered by the Parque Estadual Serra do Rola Moça conservation unit, the third largest urban park in Brazil, which is located in the transition zone between Atlantic Forest and Cerrado and has abundant natural resources. About eight kilometers west of Casa Branca is a village called Córrego do Feijão, where, in January 2019, an ore tailing dam ruptured, causing great environmental and social impact in all areas of Brumadinho municipality, as well as the death of 270 people.

Fig 1. Location of the study area in the district of Casa Branca, Brumadinho, Minas Gerais, Brazil.

Sand fly collection

Sand flies were collected bimonthly between May 2013 and July 2014, using two CDC light traps (model HP) [27], which were exposed in peridomicile areas of nine

houses (total 18 traps). The traps were exposed at 5 PM and collected after three consecutive nights at 8 AM. Collection sites with previous report of human or canine leishmaniases cases were selected. The owners of the residences where the sand flies were collected authorized verbally the installation of the traps during the period of study

Collected sand flies were taken to the laboratory where they were killed in ether, screened and clarified. The males and the females of the collections of July/2013, November/2013, March/2014 and July/2014 were mounted in Canada balsam on a glass slide for species identification. The females from the collections of May/2013, September/2013, January/2014 and May/2014 were dissected by removing the last three segments of the abdomen and head, mounted on a glass slide in Berlese liquid. The remainder of the body was individually conditioned in a sterile 1.5 mL tube for later assembly of the pools for DNA extraction.

Species identification was determined using the classification proposed by Galati [28]. The females of *Evandromyia sallesi* and *Evandromyia cortelezzii*, were identified as belonging to the cortelezzii complex because they are undistinguishable by their morphology [29]. The abbreviation of the genera followed the proposal of Marcondes [30]

Climate data

The climate data of average monthly temperature, relative humidity and rainfall, were obtained from the Automatic Weather Station of Ibirité located in the Parque Estadual Serra do Rola Moça and approximately 10 km from the study site in Casa Branca.

Statistical analyses

To evaluate species abundance the index of species abundance "ISA", and the standardized index of species abundance "SISA" were calculated [31]. The values of SISA range from 0 to 1, with values closer to 1 representing the most abundant species. The Shannon index (H) and evenness index (J) [32] were used to determine species diversity and evenness of species abundance, respectively. To assess relationships between sand fly abundance and the climate variables at Casa Branca during the study period, the Spearman correlation test was used. The DNA detection rate was calculated considering one phlebotomine sand fly infected per positive pool.

DNA extraction and identification of *Leishmania*

Females without presence of blood in their abdomen were individually stored dry at -20 °C in a 1.5 mL tube or grouped into pools of up to 20 individuals of the same species, date and site of collection. Females were subjected to DNA extraction using the Gentra Puregene® kit (Qiagen, Valencia, CA, USA) following the manufacturer's protocol.

After DNA extraction, the Polymerase Chain Reaction technique directed to the internal transcribed spacer 1 (ITS1-PCR) of *Leishmania* ribosomal DNA was performed, which amplifies a region of 300-350 bp, using the pair of primers LITSR: 5' CTGGATCATTTTCCGATG 3 'and L5.8S: 5'-TGATACCACTTATCGCACTT 3' [33, 34].

In all reaction set-ups, negative controls containing only the PCR-reagents and replacing the DNA by nuclease free water and positive controls were included: *Leishmania amazonensis* (IFLA/BR/67/PH8), *Le. braziliensis* (MHOM/BR/75/M2903), *Le. infantum* (MHOM/BR/74/PP75) and *Le. guyanensis* (MHOM/BR/75/M4147). As sand flies may host other parasites, exogenous controls have been added in PCR reactions: *Crithidia fasciculata* (FIOCRUZ-COLPROT 048), *Endotrypanum*

monterogeii (FIOCRUZ-COLPROT 151), *Herpetomonas samuelpessoai* (FIOCRUZ-COLPROT 067), *Leptomonas collosoma* (FIOCRUZ-COLPROT 073) and *Phytomonas serpens* (FIOCRUZ-COLPROT 189). These controls were kindly provided by the Coleção de Protozoários of the Laboratório de Biologia Molecular e Doenças Endêmicas (FIOCRUZ-COLPROT) of the Instituto Oswaldo Cruz, Rio de Janeiro, Brazil).

The amplicons were visualized in a 2% agarose gel stained with ethidium bromide (10mg/mL) with a 100 bp DNA Step Ladder provided as molecular length size standard. The PCR-positive products were subjected to the PCR-RFLP technique using the *Hae*III digestion enzyme as described by Schönian et al. [34].

For confirmation, all PCR amplicons were sequenced. The PCR-products were purified using the QIAquick® PCR Purification Kit (Qiagen, Valencia, CA, USA) as described by the manufacturer. The sequencing reactions were prepared in duplicate using 1 μL of the purified product, 1 μL of primer at 5 pmol (antisense and sense in separate tubes), 1 μL Big Dye Terminator v3.1 Cycle Sequencing Kit (Applied Biosystems, Foster City, CA, USA) and distilled water to a final volume of 10 μL. Sequences were then generated by the ABI 3730x1 DNA Analyzer and Geneious (v. 9.1.3) was used to check the electropherograms, align sequences and compare with the sequences deposited in the GenBank database through the BLAST alignment algorithm, considering the two most similar organisms according to identity above 90% for genus level and 98% for species level. These sequences are available in Genbank (Accession numbers MN638721-MN638737).

Ethics

The collections were carried out with the due permission of the Brazilian environmental agencies (SISBIO number 15237-2). The sand flies were deposited in the Coleção de Flebotomíneos of the Instituto René Rachou/Fiocruz (FIOCRUZ/COLFLEB).

Results

Sand fly fauna

During the study period, 16,771 sand flies of 23 species and eight genera were collected in Casa Branca: *Brumptomyia* sp., *Evandromyia cortelezzii, Ev. edwardsi, Ev. evandroi, Ev. lenti, Ev. sallesi, Ev. teratodes, Ev. termitophila, Ev. tupynambai, Lutzomyia amarali, Lu. ischyracantha, Lu. longipalpis, Migonemyia migonei, Nyssomyia intermedia, Ny. whitmani, Pintomyia bianchigalatiae, Pi. fischeri, Pi. mamedei, Pi. misionensis, Pi. monticola, Pi. pessoai, Psathyromyia pascalei,* and *Psychodopygus lloydi*.

The most abundant species was *Ny. whitmani* (70.9%; SISA 0.74), followed by *Lu. longipalpis* (15.2%; SISA 0.53) and *Mg. migonei* (9.1%; SISA 0.51) (Table 1) (Figure 2). The Shannon (H) and evenness (J) indexes were low for the study area (H'= 0.9946229; J'= 0.308997).

Fig 2. Standardized index of species abundance of species collected at Casa Branca, Brumadinho, Minas Gerais in May 2013 to July 2014.

Among the genera, *Evandromyia* was the richest with eight species, followed by *Pintomyia* with six (Table 1). The month of May 2013 showed the greatest abundance of species collected (7,054 sand flies) while July 2014 had the lowest (266). The highest species richness was observed in May 2014 with a total of 18 species (Table 1).

The climate parameters of temperature and relative humidity presented short variation during the study period. Rainfall ranged from periods of no rain in July 2013 to a maximum of 128.2 mm³ in January 2014. However, there was little difference in precipitation levels during the other months of the study (Figure 3). There is a trend of a positive correlation between the number of individuals and temperature (0.19 - Spearman correlation; p-value > 0.05). There was no correlation between the humidity and rainfall with the abundance of sand flies during the study (0.012 and -0.024 Spearman correlation, respectively; p-value > 0.05). There was no significant correlation between the number of sand flies collected each month and temperature, relative humidity or rainfall (p-value > 0.05 – Spearman correlation).

Fig 3. The relationship between seasonal variation in the number of individuals of species of sand flies collected and the climate variables at Casa Branca, Brumadinho, Minas Gerais in May 2013 to July 2014.

Molecular studies

Of a total of 4,913 females, 47 were individually tested and 4,866 grouped in 311 pools. Table 2 presents information about the species collected and their distributions for molecular analysis. *Nyssomyia whitmani* was the most abundant and therefore yielded the largest number of pools (4,128 specimens/220 pools), followed by *Lu. longipalpis* (248/19), *Mg. migonei* (144/14) and *Ps. lloydi* (115/18).

No sample individually analyzed in this study was PCR positive for *Leishmania*. A total of 13 pools were PCR-positive for *Leishmania*, being one pool of *Lu. longipalpis*, 11 of *Ny. whitmani* and one of *Ps. lloydi* (Table 3). The infection rate obtained for *Leishmania* was 0.26% (13/4,913). Twelve pools showed high similarity

values when compared to Genbank sequences (>98%), allowing us to confirm sample identity either to species or genus level; one pool of *Lu. longipalpis* presented a low similarity level (80%) to publicly available *Leishmania* sequences due to low quality of the sequences, which contained a high number of ambiguous sites.

A total of four pools of *Ny*. *whitmani* were positive for *Le*. *amazonensis* (0.08%) but it was not possible get to species level in other seven pools of this sand flies species, therefore, sequences were left as undetermined (*Leishmania* sp.) (Table 3). One pool of the species *Ps*. *lloydi* was positive for DNA of *Le*. *braziliensis* (0.02%). *Lutzomyia longipalpis* was positive for DNA of *Leishmania* sp. (0.02%) (Table 3).

Positive pools (n = 5) with a natural infection rate of 0.10% (5/4,913) for ITS1-PCR revealed the presence of bands of inconsistent size (400-500 bp) with fragments for *Leishmania* (~350 bp). These results led us to investigate the identity of these fragments and three trypanosomatid species were identified from the ITS1 fragments (Table 3).

One pool of *Ny*. *whitmani* and of cortelezzii complex were positive for *Herpetomonas* sp. One pool of *Ps. lloydi* and two pools of *Ny. whitmani* were positive for *Crithidia* sp. (Table 3). Sequence similarity to Genbank sequences ranged between 90-97%.

Discussion

The diversity of species found in this study may be explained by the fact that Casa Branca is located next to Parque Estadual Serra do Rola Moça, and so the residences selected for sampling are close to forest environments that are rich in natural resources. At the same time, the peridomicile areas of these households were conducive to the breeding and maintenance of sand fly populations including the presence of vegetation (fruit trees), the accumulation of organic matter on the ground and the

housing of domestic animals (chickens, dogs, pigs), which can serve as sources of food and shelter for sand flies.

In the present work, *Ny. whitmani* was the most abundant sand fly in the samples peridomicile areas (70.85%), being found in high density close to residences. This species has frequently been found in areas endemic for TL in southeastern Brazil, in the states of Espírito Santo, Minas Gerais and São Paulo, and is incriminated as a major vector of *Le. braziliensis* in some regions of Brazil [35, 36, 37, 38]. Furthermore, *Ny. whitmani* has also been found positive for DNA from *Le. infantum* in the state of Minas Gerais [39, 40], although nothing is known about this species role in the transmission of this species of *Leishmania*.

Lutzomyia longipalpis, the main vector of *Le. infantum* in Brazil, was the second most abundant species in Casa Branca (15.22%), and was also found to be frequent near houses. This finding shows the adaptation of this species to an environment undergoing development, which are the conditions often found among the residences of Casa Branca where dogs, chickens and pigs are being bred, and also confirm the high degree of adaptation to the anthropic environment of this species.[41, 42].

Migonemyia migonei was the third most captured species in the study area with 9.14%, and is also considered an anthropophilic species [43, 44]. In a study conducted in the state of Pernambuco, *Mg. migonei* predominantly in peridomiciliary areas and this species has been naturally infected by *Le. infantum*, suggesting that *Mg. migonei* could be serving as the vector species in endemic areas for visceral leishmaniasis where *Lu. longipalpis* is absent [45].

The genus *Evandromyia* presented the highest number of species (8). Although there are no proven vector species in this genus, natural infections or *Leishmania* DNA has been detected in individuals of some of its species. In studies conducted in the state

of Minas Gerais, Carvalho et al. [46] detected DNA of *Le. infantum* in *Ev. cortelezzii* and Saraiva et al. [47] found female of *Ev. sallesi* naturally infected by the same species of *Leishamania*. These findings indicate that the species of the cortelezzii complex (females of *Ev. cortelezzii* and *Ev. sallesi* do not differ morphologically) hold some epidemiological significance because they have been found with some frequency in the urban environment [48].

The genus *Pintomyia* was the second richest, with six species. Some species of this genus have been shown to frequent peridomicile areas in association with domestic animal shelters, and it is known to be anthropophilic [43]. In addition, some species have been found with *Leishmania* DNA in Brazil, thus being associated with transmission of the disease in endemic areas [49, 50, 51].

The highest standardized index of species abundance (SISA) in this study was found for *Ny. whitmani* (0.74). Regarding diversity and evenness, despite the study area being quite diverse (H '= 0.995), the abundances of the species were not similar, with most individuals belonging to the dominant species *Ny. whitmani*. This likely explains the low value for the evenness index (J '= 0.309), since the great abundance of one species tends to cause less uniformity among the others.

The correlations detected between seasonal variation in sand fly abundance and climate data (temperature, humidity and rainfall) did not reach statistical significance according to the Spearman correlation test. This can be explained by the small amount of variation in relative humidity, temperature and rainfall of each month during the study period, and a greater amount of variation may be needed to influence the population dynamics of sand flies. Furthermore, other factors not tested could be responsible for the variation in the number of sand flies collected each month, such as changes at sites throughout the year such as the presence of hosts, the type of

vegetation, cleanliness of sites or even microclimate characteristics. All of these factors could influence the sand fly populations throughout the year in Casa Branca.

Another important factor is the study period. It is widely known that to ensure greater reliability of seasonal data it is desirable that a study encompass at least two consecutive years so that they can be compared and reduce the error caused by atypical climate conditions that may be occur throughout the year. So the number of bimonthly collections may not have been sufficient to ascertain the veracity the relationships of temperature, humidity and rainfall with the number of individual sand flies collected.

With regard to the feeding habit of sand fly species found in the Casa Branca District, it is interesting to highlight the study carried out in the same area by Carvalho et al. [52] who showed that several of them are eclectic about eating habits. *Nyssomyia whitmani* and *Mg. migonei* fed more frequently on birds, followed by dogs and *Lu. longipalpis* in humans followed by dogs.

Nyssomyia whitmani-pools were positive for *Le. amazonensis*. Changes in the natural environment may have triggered a new epidemiological profile of leishmaniases that could involve *Leishmania amazonensis*, an alternative vector (i.e. *Ny. whitmani*) and new reservoirs (domestic and wild). Similar fact has been reported in the literature for other species of sand flies [53, 54]. Added to this, in an experimental infection study conducted by Fonteles et al. [55] it was possible to infect *Ny. whitmani* with *Le. amazonensis*, showing that this sand fly species has the potential to become infected by other *Leishmania* species, being considered as a probable vector of *Le. amazonensis* in the state of Maranhão. In view of this, *Ny. whitmani* could be act in the transmission cycle of *Le. amazonensis* in Casa Branca.

The species *Ps. lloydi* has been associated with the transmission of *Le. braziliensis* in wild areas of Minas Gerais [56, 57] and was also found in this state with DNA of *Le.*

infantum [58]. *Psychodopygus lloydi* was positive for DNA of *Leishmania braziliensis*, suggesting that this sand fly could maintain a wild cycle of TL in the District of Casa Branca.

We found three species of sandflies with DNA from trypanosomatids other than *Leishmania*: *Ny*. *whitmani* with *Herpetomonas* sp. (two pools) and *Crithidia* sp. (one pool); *Ps*. *lloydi* with a pool of *Crithidia* sp. and a pool of the cortelezzii complex with Herpetomonas sp. This record is important, since recently other trypanosomatids have been found in humans [59, 60]

Only the PCR-RFLP technique using the restriction enzyme *Hae III* did not prove to be a good tool to differentiate the species of the *Leishmania* genus from other trypanosomatids detected in the pools of sand flies females in this paper. However, when associated with sequencing, the technique proved to be effective for specific identification of trypanosomatids. This fact can be explained by the molecular target used (ITS1) that does not distinguish in a same pool *Leishmania* species from other trypanosomatids, generating overlapping restriction profiles that made it difficult to identify the species, since the PCR-RFLP technique was initially described to generate restriction profiles for species of the genus *Leishmania* in clinical samples of patients, and not for the other trypanosomatids [34]. Thus, the use of this technique did not produce a satisfactory result when used for characterization of other trypanosomatids (non-*Leishmania*) detected in sand flies.

Currently, molecular techniques for the detection of trypanosomatids are being widely used. The combination of molecular techniques allows us to determine the species of the trypanosomatid. The use of the PCR technique directed to the mini-circle of kDNA demonstrated the presence of *Herpetomonas samuelpessoai* in *Pintomyia fischeri* in the State of Espírito Santo [50], and in a study carried out in Brasília using

different molecular markers, trypanosomatid DNA from the genus *Blastocrithidia* was found for the first time in *Ny*. *whitmani* and *Trypanosoma* sp. in *Evandromyia evandroi* [14]. The use of the ITS1 target allowed the detection of *Crithidia fasciculata* in sand flies in Cerrado area in the state of Minas Gerais [15]. Although these findings demonstrate the presence of trypanosomatid DNA in sand flies in Brazil, further studies are needed to clarify the associations of sand flies with trypanosomatid species.

DNA sequencing has generated good results when used for detection of *Leishmania* in sand flies [22, 61]. However, there are limitations to the use of ITS1 for *Leishmania* detection in sand flies, such as: i. the high number of copies of ITS1 yields poor quality sequencing reads; ii. ITS1 is highly conserved and has been amplifying fragments of other trypanosomatid species in field samples; iii. lower taxonomic resolution for species such as *Le. braziliensis* and *Le. guyanensis*. Thus, the final result depends on the quality of the sample, along with molecular biology techniques (DNA extraction, PCR, sequencing and phylogenetic analyzes). Despite these limitations, our results allow the identification of other species of trypanosomatids in addition to the *Leishmania* genus in sand flies.

Conclusion

This is the first study of a sand fly fauna in the municipality of Brumadinho, Minas Gerais. Our findings show that the Casa Branca locality has a diverse sand fly fauna with species that have been previously reported in the state of Minas Gerais. The *Ny*. *whitmani* species is the probable vector of *Le*. *braziliensis* in Casa Branca, and may also be involved in the transmission of *Le*. *amazonensis*.

The knowledge of the interactions between sand flies and the species of *Leishmania* and other trypanosomatids detected in this study shows that the infection may be occurring in the peridomiciliary environment in the study area. In addition,

these results help to understand the dynamics of the leishmaniases transmission cycle in Casa Branca providing support for disease control actions in the region. The large number of pools of infected sand flies raises an issue that has been neglected and is of great importance, which would be the circulation of these parasites among phlebotomine insects, and that some molecular techniques can detect the presence of these parasites in sand flies, requiring attention to interpretation results.

Acknowledgments: We thank Surveillance System in Health Brumadinho for providing epidemiological data and we thank the residents of the collection sites for making the completion of the work possible.

References

1. Alvar J, Vélez ID, Bern C, Herrero M, Desjeux P, et al. Leishmaniasis Worldwide and Global Estimates of Its Incidence. PLoS ONE 2012; 7(5): e35671.
2. Alkan C, Moin Vaziri V, Ayhan N, Badakhshan M, Bichaud L, Rahbarian N, et al. Isolation and sequencing of Dashli virus, a novel Sicilian-like virus in sandflies from Iran; genetic and phylogenetic evidence for the creation of one novel species within the *Phlebovirus* genus in the *Phenuiviridae* family. PLoS Negl Trop Dis. 2017; 11: e0005978.
3. Lestinova T, Vlkova M, Votypka J, Volf P, Rohousova I. *Phlebotomus papatasi* exposure cross-protects mice against *Leishmania major* co-inoculated with *Phlebotomus duboscqi* salivary gland homogenate. Acta Trop. 2015; 144: 9–18.

4. Brazil RP, Rodrigues AAF, Andrade-Filho JD. Sand Fly Vectors of Leishmania in the Americas - A Mini Review. Entomol Ornithol Herpetol Curr Res. OMICS International. 2014; 4: 144.

5. Shimabukuro PHF, Andrade AJ, Galati EAB. Phlebotominae in Taxonomic Catalog of the Brazilian Fauna. PNUD. 2018. Available from: http://fauna.jbrj.gov.br/fauna/faunadobrasil/3297.

6. Deane LM, Deane MP. Visceral leishmaniasis in Brazil: geographical distribution and transmission. Rev Inst Med Trop São Paulo. 1962; 4: 198-212.

7. Lainson R, Shaw JJ. The role of animals in the epidemiology of South American leishmaniasis. In: Lumsden EDA, editor. Biology of the Kinetoplastida. London and New York: Academic Press. 1979; 2: 1-116.

8. Mayrink W, Williams P, Coelho M V, Dias M, Martins AV, Magalhães PA, et al. Epidemiology of dermal leishmaniasis in the Rio Doce Valley, State of Minas Gerais, Brazil. Ann Trop Med Parasitol. 1979; 73: 123-137.

9. Andrade Filho JD, Galati EAB, Falcão AL. *Nyssomyia intermedia* (Lutz & Neiva, 1912) and *Nyssomyia neivai* (Pinto, 1926) (Diptera: Psychodidae: Phlebotominae) geographical distribution and epidemiological importance. Mem Inst Oswaldo Cruz. Fundação Oswaldo Cruz; 2007; 102: 481-487.

10. Christensen HA, Herrer A. Neotropical Sand Flies (Diptera: Psychodidae), Invertebrate Hosts of *Endotrypanum Schaudinni* (Kinetoplastida: Trypanosomatidae). J Med Entomol. 1976; *13*: 299-303.

11. Kalantari M, Motazedian MH, , Soltani Z, Soltani A, Azizi K. Bionomics of phlebotomine sand flies species (Diptera: Psychodidae) and their natural infection with *Leishmania* and *Crithidia* in Fars province, southern Iran Mohsen. J Parasit Dis. 2018; 42: 511-518

12. Kato H, Uezato H, Sato H, Bhutto AM, Soomro FR, Baloch JH, et al. Natural infection of the sand fly *Phlebotomus kazeruni* by Trypanosoma species in Pakistan. Parasites & Vectors. 2010; 3: 10.

13. Nzelu CO, Kato H, Puplampu N, Desewu K, Odoom S, Wilson MD, et al. First Detection of *Leishmania tropica* DNA and *Trypanosoma* Species in *Sergentomyia* Sand Flies (Diptera: Psychodidae) from an Outbreak Area of Cutaneous Leishmaniasis in Ghana. PLoS Negl Trop Dis. 2014; 8: 2630.

14. Ferreira TS, Minuzzi-Souza TTC, Andrade AJ, Coelho TO, Rocha DA, Obara MT, et al. Molecular detection of *Trypanosoma* sp. and *Blastocrithidia* sp. (Trypanosomatidae) in phlebotominesand flies (Psychodidae) in the Federal District of Brazil. Rev Soc Bras Med Trop. 2015; 48: 776-779.

15. Saraiva L, Reis AS, Rugani JMN, Pereira AAS, Rêgo FD, Lima ACVMR, et al. Survey of sand flies (Diptera: Psychodidae) in an environmentally protected area in Brazil. Plos One. 2015; 10: 1-5.

16. Espinosa OA, Serrano MG, Camargo EP, Teixeira MM, Shaw JJ. An appraisal of the taxonomy and nomenclature of trypanosomatids presently classified as *Leishmania* and *Endotrypanum*. Parasitology. 2016; 15 :1-13.

17. Harhay MO, Olliaro PL, Costa DL, Costa CHN. Urban parasitology: visceral leishmaniasis in Brazil. Trends Parasitol. 2011; 27: 403-409.

18. Silva TAM, Coura-Vital W, Barbosa DS, Oiko CSF, Morais MHF, Tourinho BD, et al. Spatial and temporal trends of visceral leishmaniasis by mesoregion in a southeastern state of Brazil, 2002-2013. PLoS Negl Trop Dis. 2017; 11: e0005950.

19. Gontijo CMF, Silva ES, Fuccio MB, Sousa MCA, Pacheco RS, Dias ES, et al. Epidemiological studies of an outbreak of cutaneous leishmaniasis in the Rio Jequitinhonha Valley, Minas Gerais, Brazil. Acta Trop. 2002; 81: 143-150.

20. Prado PF, Rocha MF, Sousa JF, Caldeira DI, Paz GF, Dias ES. Epidemiological aspects of human and canine visceral leishmaniasis in Montes Claros, State of Minas Gerais, Brazil, between 2007 and 2009. Rev Soc Bras Med Trop. 2011; 44: 561-566.

21. Barata RA, Peixoto JC, Tanure A, Gomes ME, Apolinário EC, Bodevan EC, et al. Epidemiology of visceral leishmaniasis in a reemerging focus of intense transmission in Minas Gerais State, Brazil. Biomed Res Int. 2013; 2013 Article ID 405083

22. Saraiva L, Leite CG, Lima AC, Carvalho LO, Pereira AA, Rugani JM, et al. Seasonality of sand flies (Diptera: Psychodidae) and *Leishmania* DNA detection in vector species in an area with endemic visceral leishmaniasis. Mem Inst Oswaldo Cruz. 2017; 112: 309-318.

23. Saraiva L, Andrade Filho JD, Falcão AL, de Carvalho DAA, de Souza CM, Freitas CR, et al. Phlebotominae fauna (Diptera: Psychodidae) in an urban district of Belo Horizonte, Brazil, endemic for visceral leishmaniasis: Characterization of favored locations as determined by spatial analysis. Acta Trop. 2011; 117: 137-145.

24. Souza CM, Pessanha JE, Barata RA, Monteiro EM, Costa DC, Dias ES. Study on Phlebotomine Sand Fly (Diptera: Psychodidae) Fauna in Belo Horizonte, State of Minas Gerais, Brazil. Mem Inst Oswaldo Cruz. 2004; 99: 795-803.

25. Pereira AAS, Ferreira EC, Lima ACVMR, Tonelli GB, Rêgo FD, Paglia AP, et al. (2017) Detection of *Leishmania* spp in silvatic mammals and isolation of *Leishmania*(*Viannia*) *braziliensis* from *Rattus rattus* in an endemic area for leishmaniasis in Minas Gerais State, Brazil. PLoS ONE. 2017; 12: e0187704.

26. IBGE. Estimativa da População, Brumadinho, Minas Gerais, Brasil. Instituto Brasileiro de Geografia e Estatística (IBGE). 2016. http://www.cidades.ibge.gov.br/xtras/perfil.php?lang=&codmun=310900. Accessed 20 Jul 2016.

27. Pugedo H, Barata RA, França-Silva JC, Silva JC, Dias ES. HP: um modelo aprimorado de armadilha luminosa de sucção para a captura de pequenos insetos. Rev Soc Bras Med Trop. 2005; 38: 70-72.

28. Galati EAB. Phlebotominae (Diptera, Psychodidae): Classification, morphology, and terminology of adults and identification of American taxa In: Rangel E.F. & Shaw J.J. (eds.) Brazilian sand flies: Biology, taxonomy, medical importance and control. Rio de Janeiro, Brazilian Ministry of Health. Oswaldo Cruz Foundation, 2018, pp.9–212.

29. Szelag ESA, Rosa JR, Galati EAB, Andrade Filho JD, Salomón OD.Considerations on the species complex of the Cortelezzii series (Diptera: Psychodidae) and description of *Evandromyia chacuensis* sp. nov., a new phlebotomine species of the Chaco Region, Argentina**.** J Med Entomol. 2018; 55: 902–909.

30. Marcondes CB. A proposal of generic and subgeneric abbreviations for phlebotomine sandflies (Diptera: Psychodidae: Phlebotominae) of the world. Entomol News. 2007; 118: 351-356.

31. Roberts DR, Hsi BP. An Index of Species Abundance for Use with Mosquito Surveillance Data. Environ Entomol. 1979; 8: 1007-1013.

32. Hayek LAC, Buzas MA. Surveying Natural Populations. New York, Columbia University Press. 1997: 347-389.

33. El Tai N O, Osman OF, El Fari M, Presber W, Schönian G. Genetic heterogeneity of ribosomal internal transcribed spacer (its) in clinical samples of *Leishmania donovani* spotted on filter paper as revealed by single-strand conformation polymorphisms (sscp) and sequencing. Trans Royal Soc Trop Med Hyg. 2000; 94: 1-5.

34. Schönian G, Nasereddin A, Dinse N, Schweynoch C, Schallig HD, Presber W et al. PCR diagnosisand characterization of *Leishmania* in local and imported clinical samples. Diagn Microbiol Infect Dis. 2003; 47: 349-358.

35. Colla-Jacques FE, Casanova C, Prado AP. Study of sand fly fauna in an endemic area of American cutaneous leishmaniasis and canine visceral leishmaniasis in the municipality of Espírito Santo do Pinhal, São Paulo, Brazil. Mem Inst Oswaldo Cruz. 2010; 105): 208-215.

36. Costa SM da, Cechinel M, Bandeira V, Zannuncio JC, Lainson R, Rangel EF. *Lutzomyia* (*Nyssomyia*) *whitmani* s.l . (Antunes & Coutinho, 1939) (Diptera: Psychodidae: Phlebotominae): geographical distribution and the epidemiology of American cutaneous leishmaniasis in Brazil Mini-review. Mem Inst Oswaldo Cruz. Fundação Oswaldo Cruz. 2007; 102: 149-153.

37. Virgens TM, Santos CB, Pinto IS, Silva KS, Leal FC, Falqueto A. Phlebotomine sand flies (Diptera, Psychodidae) in an American tegumentary leishmaniasis transmission area in northern Espírito Santo State, Brazil. Cad Saude Publica. 2008; 24: 2969-2978.

38. Souza CF, Quaresma PF, Andrade Filho JD, Bevilacqua PD. Phlebotomine fauna in the urban area of Timóteo, State of Minas Gerais, Brazil. Acta Trop. 2014; 134: 72-79.

39. Saraiva L, Andrade Filho JD, Silva SO, Andrade ASR, Melo MN. The molecular detection of different *Leishmania* species within sand flies from a cutaneous and visceral leishmaniasis sympatric area in Southeastern Brazil. Mem Inst Oswaldo Cruz.2010; 105: 1033-1039.

40. Margonari C, Soares RP, Andrade-Filho JD, Xavier DC, Saraiva L, Fonseca AL, et al. Phlebotomine Sand Flies (Diptera: Psychodidae) and *Leishmania* Infection in Gafanhoto Park, Divinópolis, Brazil. J Med Entomol. 2010; 47: 1212-1219.

41. Macedo-Silva VP, Martins DRA, De Queiroz PVS, Pinheiro MPG, Freire CCM, Queiroz JW, et al. Feeding Preferences of *Lutzomyia longipalpis* (Diptera: Psychodidae), the Sand Fly Vector for *Leishmania infantum* (Kinetoplastida: Trypanosomatidae). J Med Entomol. 2014; 51: 237-244.

42. Salomón OD, Feliciangeli MD, Quintana MG, Afonso MM dos S, Rangel EF. *Lutzomyia longipalpis* urbanisation and control. Mem Inst Oswaldo Cruz. 2015; 110: 831-846.

43. Gomes AC, Barata JMS, Rocha e Silva EO, Galati EAB. Aspectos ecológicos da leishmaniose tegumentar americana. 6. Fauna flebotomínea antropófila de matas residuais situadas na região centro-nordeste do Estado de são paulo, brasil. Rev Inst Med Trop São Paulo. 1989; 31: 32-39.

44. Moschin JC, Ovallos FG, Sei IA, Galati EAB, Moschin JC, Ovallos FG, et al. Ecological aspects of phlebotomine fauna (Diptera, Psychodidae) of Serra da Cantareira, Greater São Paulo Metropolitan region, state of São Paulo, Brazil. Rev Bras Epidemiol. 2013; 16: 190-201.

45. Carvalho MR, Valença HF, Silva FJ, Pita-Pereira D, Araújo Pereira T, Britto C, et al. Natural *Leishmania infantum* infection in *Migonemyia migonei* (França, 1920) (Diptera: Psychodidae: Phlebotominae) the putative vector of visceral leishmaniasis in Pernambuco State, Brazil. Acta Trop. 2010; 116: 108-110.

46. Carvalho GML, Andrade Filho JD, Falcão AL, Lima ACVMR, Gontijo CMF. Naturally Infected *Lutzomyia* Sand Flies in a Leishmania- Endemic Area of Brazil. Vector-Borne Zoonotic Dis. 2008; 8: 407-414.

47. Saraiva L, Carvalho GML, Quaresma PF, Rocha Lima ACVM, Falcão AL, Andrade-Filho JD. Natural infection of *Nyssomyia neivai* (Pinto, 1926) and *Evandromyia sallesi* (Galvão & Coutinho, 1939) (Diptera: Psychodidae) by *Leishmania infantum chagasi* (Cunha & Chagas, 1937) in Minas Gerais, Brazil. J Med Entomol. 2009; 46: 1159-1163.

48. Lana RS, Michalsky EM, Fortes-Dias CL, França-Silva JC, Lara-Silva FO, Lima ACVMR, Avelar DM, Martins JCD, Dias ES. Phlebotomine Sand Fly Fauna and Leishmania Infection in the Vicinity of the Serra do Cipó National Park, a Natural Brazilian Heritage Site. BioMed Res Int. 2015; 385493

49. Silva OS, Grunewald J. Contribution to the sand fly fauna (Diptera: Phlebotominae) of Rio Grande do Sul, Brazil and *Leishmania* (*Viannia*) infections. Mem Inst Oswaldo Cruz. 1999; 94: 579-582.

50. Rocha LS, Santos CB, Falqueto A, Grimaldi G, Cupolilo E. Molecular biological identification of monoxenous trypanosomatids and *Leishmania* from antropophilic sand flies (Diptera: Psychodidae) in Southeast Brazi. Parasilto Res. 2010; 107: 465-468.

51. Rêgo FD, Souza GD, Dornelles LFP, Andrade Filho JD. Ecology and Molecular Detection of *Leishmania infantum* Nicolle, 1908 (Kinetoplastida:

Trypanosomatida) in Wild-Caught Sand Flies (Psychodidae: Phlebotominae) Collected in Porto Alegre, Rio Grande do Sul: A New Focus of Visceral Leishmaniasis in Brazil. J Med Entomol 2019; 56: 519–525.

52. Carvalho, GML, Rêgo FD, Tanure A, Silva ACP, Dias TA, Paz GF, et al. Blood Meal Identification in Field-Captured Sand flies: cytochrome *b* PCR followed by DNA sequencing technique. J Med Entomol. 2017; 54: 1049-1054

53. Loiola CF, Silva DA, Galati EAB. Phlebotomine fauna (Diptera: Psychodidae) and species abundance in an endemic area of American cutaneous leishmaniasis in southeastern Minas Gerais, Brazil. Mem Inst Oswaldo Cruz. 2007; 102: 581-585.

54. Colla-Jacques FE, Casanova C, Prado AP. Study of sand fly fauna in an endemic area of American cutaneous leishmaniasis and canine visceral leishmaniasis in the municipality of Espírito Santo do Pinhal, São Paulo, Brazil. Mem Inst Oswaldo Cruz. 2010; 105: 208-215.

55. Fonteles RS, Pereira Filho AA, Moraes JL, P , Kuppinger O, and Rebêlo JMM. Experimental infection of *Lutzomyia (Nyssomyia) whitmani* (Diptera: Psychodidae: Phlebotominae) with *Leishmania* (*Viannia*) *braziliensis* and *Leishmania* (*L.*) *amazonensis*, etiological agents of American Tegumentary Leishmaniasis. J Med Entomol. 2016; 53: 206-209.

56. Quaresma, P. F., G. M. L. Carvalho, M. C. N. F. Ramos, and J. D. Andrade Filho. 2012. Natural *Leishmania* sp. reservoirs and phlebotomine sandfly food source identification in Ibitipoca State Park, Minas Gerais, Brazil. Mem Inst Oswaldo Cruz. 107: 480-485.

57. Tonelli GB, Tanure A, Rêgo FD, Carvalho GML, Simões TC, Andrade Filho JD. Aspects of the ecology of phlebotomine sand flies (Diptera: Psychodidae) in

the Private Natural Heritage Reserve Sanctuary Caraça. PLoS ONE 2017; 12: e0178628.

58. Lara-Silva FO, Michalskya, Fortes-Dias CL, Fiuza VOL, Pessanha JEM, Regina-Silva S. Epidemiological aspects of vector, parasite, and domestic reservoir in areas of recent transmission and no reported human cases of visceral.

59. Maruyama SR, Santana AKM, Takamiya NT, Takahashi TY, Rogerio LA, Oliveira CAB, et al. Non-*Leishmania* parasite in fatal visceral leishmaniasis–like disease, Brazil. Emerg Infect Dis. 2019;25: 2088–2092.

60. Ghobakhloo N, Motazedian MH, Naderi S, Sepideh E. Isolation of *Crithidia* spp. from lesions of immunocompetent patients with suspected cutaneous leishmaniasis in Iran. Trop Med Int Health. 2019; 24: 116–126.

61. Rêgo FD, Rugani JMN, Shimabukuro PHF, Tonelli GB, Quaresma PF, Gontijo CMF. Molecular detection of *Leishmania* in Phlebotomine sand flies (Diptera: Psychodidae) from a cutaneous leishmaniasis focus at Xakriabá Indigenous Reserve, Brazil. PLoS One; 2015. 10: e0122038.

Supporting Information Legends

S1 License for the collection of sandflies granted by the Brazilian environmental agency (SISBIO)

S2 The copyright permission for figure 1.

S3 Revision English by native speaker

Table 1. Sand flies collected according to month at the Casa Branca, Brumadinho, Minas Gerais, from May 2013 to July 2014.

Species	May/13 ♂	May/13 ♀	Jul/14 ♂	Jul/14 ♀	Sep/13 ♂	Sep/13 ♀	Nov/13 ♂	Nov/13 ♀	Jan/14 ♂	Jan/14 ♀	Mar/14 ♂	Mar/14 ♀	May/14 ♂	May/14 ♀	Jul/14 ♂	Jul/14 ♀	Total ♂	Total ♀	Total (%)
Brumptomyia sp.	0	0	0	1	4	4	0	0	0	0	0	2	0	0	0	0	4	7	11 (0.1)
Cortelezzii complex	0	7	0	2	0	12	0	5	0	1	0	1	0	9	0	1	0	38	38 (0.2)
Evandromyia cortelezzii	1	0	2	0	3	0	1	0	2	0	1	0	0	0	0	0	10	0	10 (0.1)
Evandromyia edwarsi	0	0	0	0	0	0	0	0	0	0	0	0	0	1	0	0	0	1	1 (0.0)
Evandromyia evandroi	0	0	0	0	0	0	0	0	1	0	0	0	0	0	0	0	1	0	1 (0.0)
Evandromyia lenti	1	0	0	1	2	2	10	5	39	29	0	2	2	3	0	1	54	43	97 (0.6)
Evandromyia sallesi	1	0	0	0	2	0	0	0	0	0	0	0	1	0	1	0	5	0	5 (0.0)
Evandromyia teratodes	0	0	0	0	0	0	0	0	0	0	0	0	0	1	0	0	0	1	1 (0.0)
Evandromyia termitophila	0	1	0	1	0	1	0	5	0	0	0	0	0	3	0	0	0	11	11 (0.1)
Evandromyia tupynambai	0	1	0	0	3	0	0	2	0	2	0	0	1	10	0	0	4	15	19 (0.1)
Lutzomyia amarali	0	0	0	0	0	0	0	0	0	0	0	2	0	1	0	0	0	3	3 (0.0)
Lutzomyia ischyracantha	0	0	0	0	1	0	0	0	0	0	0	0	0	0	0	0	1	0	1 (0.0)
Lutzomyia longipalpis	528	127	57	5	430	112	181	41	63	22	214	62	547	104	55	3	2,075	476	2551 (15.2)
Migonemyia migonei	219	90	72	12	175	53	156	49	104	17	73	24	373	62	41	13	1213	320	1533 (9.1)
Nyssomyia intermedia	0	0	1	2	1	0	0	0	0	0	0	0	0	0	0	0	2	2	4 (0.0)
Nyssomyia whitmani	3,449	2,535	79	263	793	680	547	343	625	324	101	72	974	959	77	62	6,645	5,238	11,883 (70.9)
Pintomyia bianchigalatiae	0	13	0	0	0	10	1	2	0	3	0	17	0	30	0	0	1	75	76 (0.5)
Pintomyia fischeri	7	13	6	2	7	11	34	15	27	34	14	18	11	49	5	4	111	146	257 (1.5)
Pintomyia mamedei	0	1	0	0	0	0	0	0	0	0	0	0	0	0	0	0	0	1	1 (0.0)
Pintomyia misionensis	0	0	0	3	0	0	0	0	0	1	0	0	0	4	0	1	0	9	9 (0.1)
Pintomyia monticola	1	4	0	2	0	1	2	0	0	1	0	6	2	21	1	0	6	35	41 (0.2)
Pintomyia pessoai	1	1	0	0	1	18	0	19	3	0	1	0	1	3	0	0	7	41	48 (0.3)
Psathyromyia pascalei	0	0	0	0	1	0	0	0	0	1	0	0	1	0	1	0	3	1	4 (0.0)
Psathyromyia sp.	0	0	0	1	0	0	0	0	0	0	0	0	0	0	0	0	0	1	1 (0.0)
Psychodopygus lloydi	1	33	0	4	0	0	6	3	4	47	0	0	6	38	0	0	17	125	142 (0.9)
**Total**	4209	2845	217	299	1423	908	938	489	868	482	404	206	1919	1298	181	85	10159	6612	16771 (100)
	**7,054**		**516**		**2,331**		**1427**		**1,350**		**610**		**3,217**		**266**		**16,771**		

Table 2. Sand fly organized by species that were analyzed individually and in pools collected in Casa Branca, Brumadinho, Minas Gerais, from May 2013 to May 2014.

Species	Sand flies collected	number of analyzed pools	specimens analyzed individually
Brumptomyia sp*	4	1	0
Cortelezzii complex	25	5	3
Evandromyia edwardsi	1	0	1
Evandromyia lenti	31	4	3
Evandromyia teratodes	1	0	1
Evandromyia termitophila	6	1	4
Evandromyia tupynambai	13	2	1
Lutzomyia amarali	1	0	1
Lutzomyia longipalpis	248	19	0
Migonemyia migonei	144	14	0
Nyssomyia whitmani	4128	220	0
Pintomyia bianchigalatiae	47	8	2
Pintomyia fischeri	80	13	0
Pintomyia misionensis	4	0	4
Pintomyia monticola	27	3	3

Pintomyia pessoai	16	3	1
Pintomyia sp.*	21	0	21
Psathyromyia pascalei	1	0	1
Psychodopygus lloydi	115	18	1
**Total**	**4913**	**311**	**47**

* sand flies damaged

Table 3. Results of DNA detection and identification of species of Trypanosomatidae according to species of sand fly, number of pool and collection date in Casa Branca, Brumadinho from May 2013 to May 2014.

Accession numbers	% similarity	Seq ID Blast	Host	Collection date
MN638721	97,31	*Crithidia* sp.	*Nyssomyia whitmani*	may-2013
MN638722	95,38	*Herpetomonas* sp.	Complexo cortelezzii	may-2013
MN638723	90,21	*Herpetomonas* sp.	*Nyssomyia whitmani*	may-2013
MN638724	99,51	*Leishmania* sp.	*Nyssomyia whitmani*	may-2013
MN638725	99,6	*Leishmania* sp.	*Nyssomyia whitmani*	may-2013
MN638726	100	*Leishmania amazonensis*	*Nyssomyia whitmani*	may-2013
MN638727	100	*Leishmania amazonensis*	*Nyssomyia whitmani*	may-2013
MN638728	96,5	*Crithidia* sp.	*Psychodopygus lloydii*	may-2014
MN638729	97	*Herpetomonas* sp.	*Nyssomyia whitmani*	may-2013
MN638730	99,32	*Leishmania braziliensis*	*Psychodopygus lloydii*	may-2014
MN638731	99,66	*Leishmania* sp.	*Nyssomyia whitmani*	may-2013
MN638732	99,32	*Leishmania* sp.	*Nyssomyia whitmani*	may-2013
MN638733	80,2	*Leishmania* sp.	*Lutzomyia longipalpis*	may-2013
MN638734	98,01	*Leishmania amazonensis*	*Nyssomyia whitmani*	sep-2013
MN638735	99,04	*Leishmania* sp.	*Nyssomyia whitmani*	may-2013
MN638736	98,45	*Leishmania* sp.	*Nyssomyia whitmani*	may-2013
MN638737	98,72	*Leishmania* sp.	*Nyssomyia whitmani*	may-2013
MN638738	99,39	*Leishmania amazonensis*	*Nyssomyia whitmani*	may-2013

Legend: Seq ID Blast = hit provided by Blast search.

[1] Trypanosomatid sequence obtained from strain A9 from the Coleção de Protozoários (FIOCRUZ/COLPROT).

Supporting Information

Fig. 1 - Location of Casa Branca district on the map of the state of Minas Gerais, Brazil.

Fig. 2 - Standardized index of species abundance of species collected at Casa Branca, Brumadinho, Minas Gerais between May 2013 and July 2014.

Fig. 3 - The relationship between seasonal variation in the number of individuals of species of sand flies collected and the climate variables at Casa Branca, Brumadinho, Minas Gerais in May 2013 to July 2014.

Figure 1

Click here to access/download;Figure;Fig. 1.tif

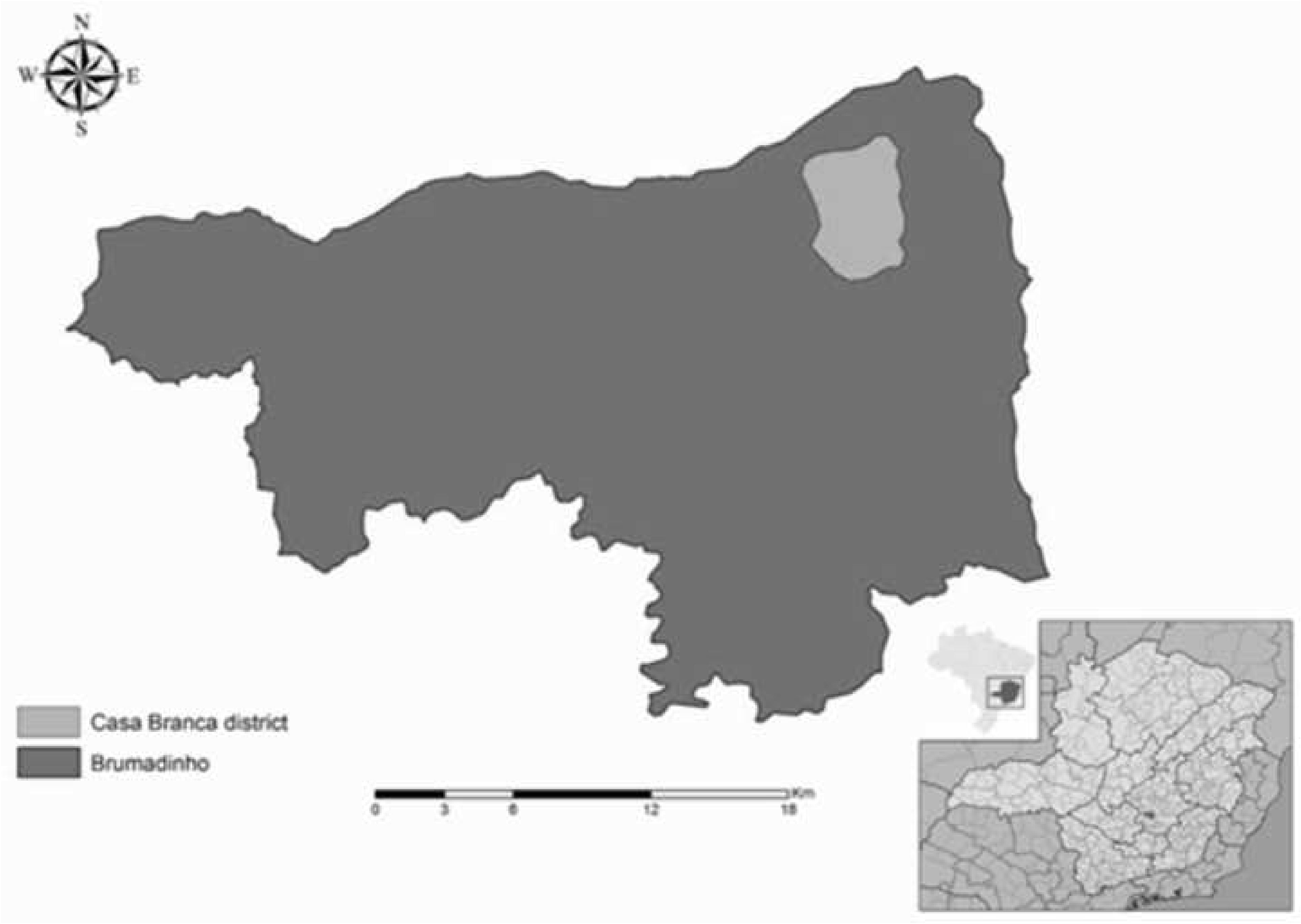

Figure 2

Click here to access/download;Figure;Fig. 2.tif

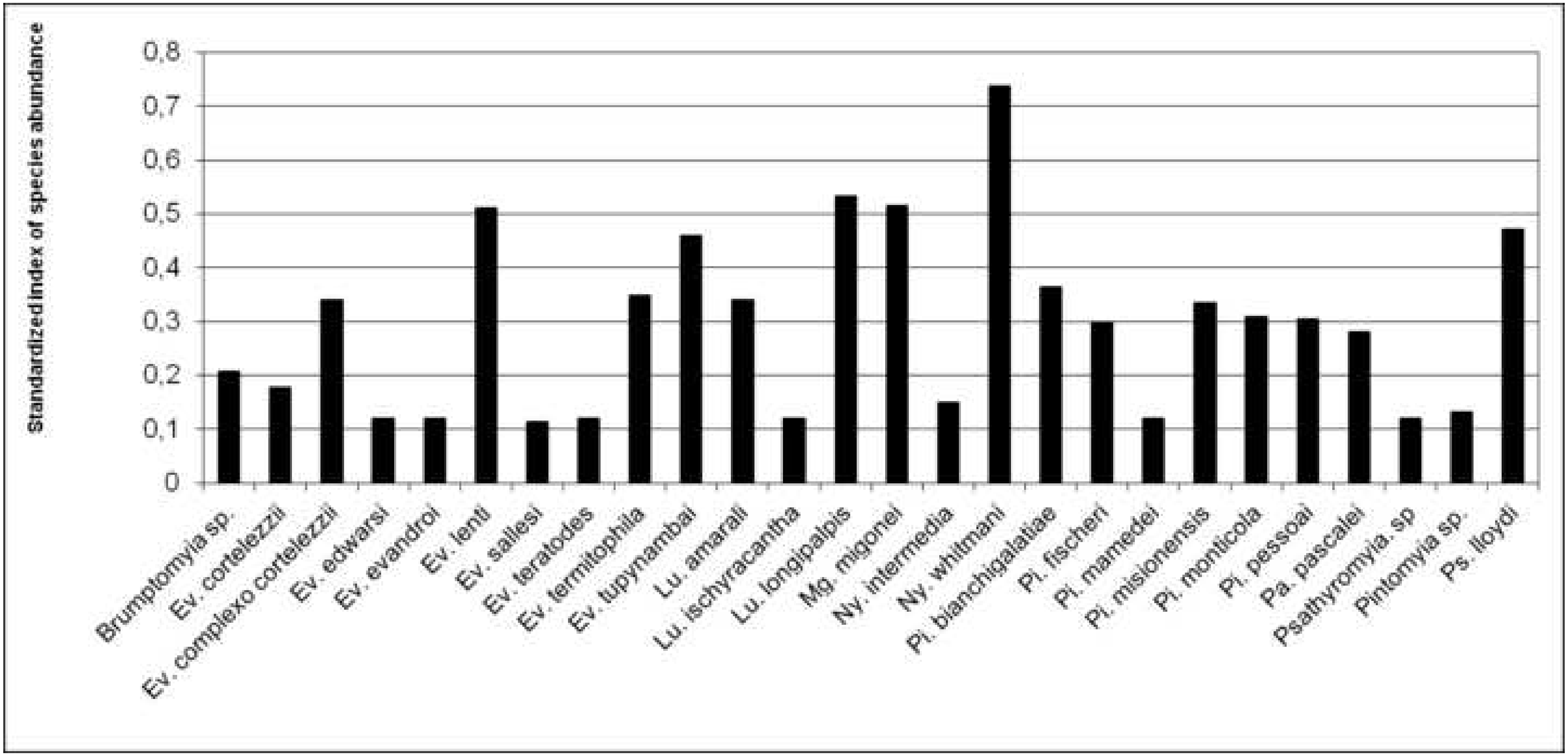

Figure 3

Click here to access/download;Figure;Fig. 3.tif

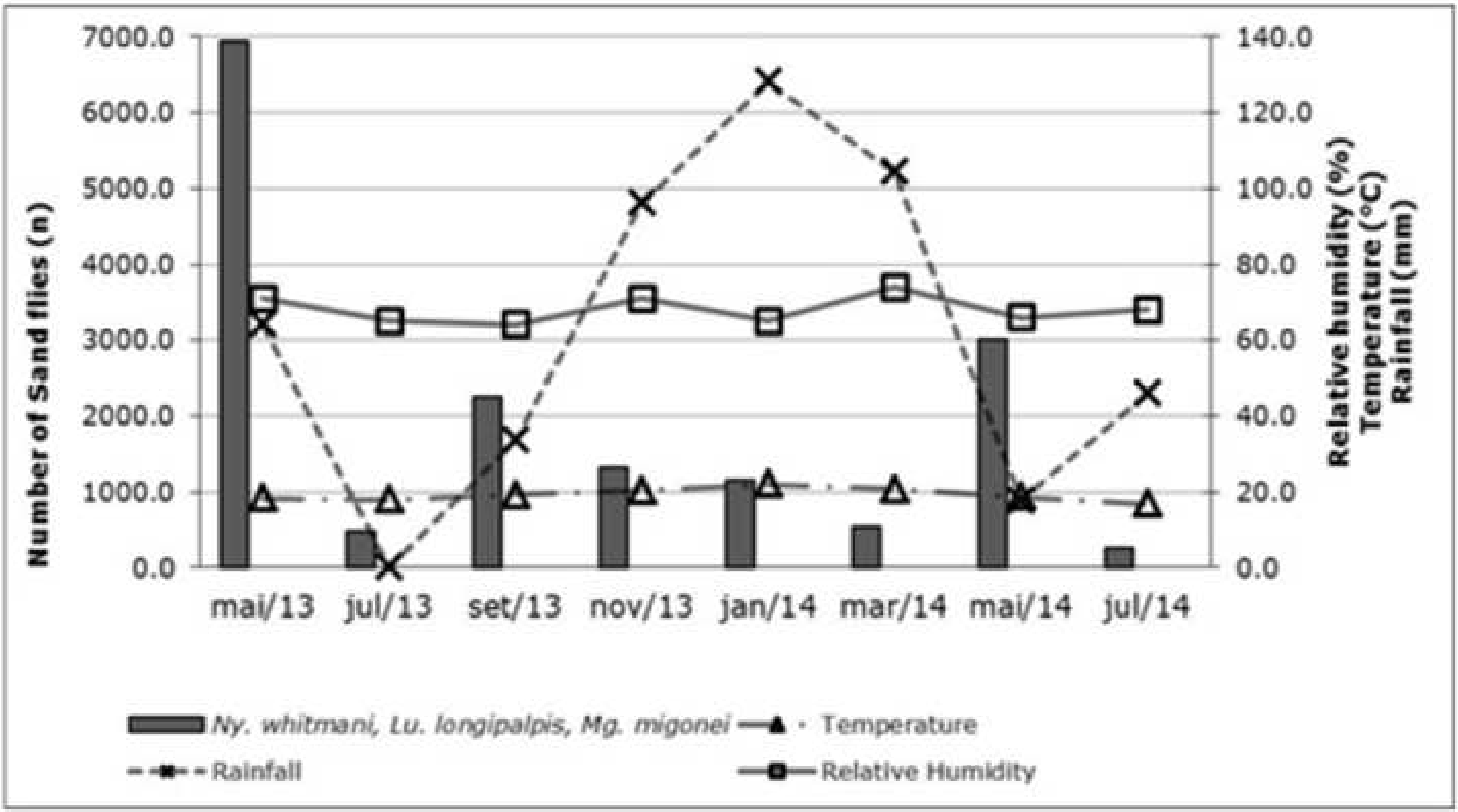

Short title: Phlebotomine sand flies and trypanosomatids in area of leishmaniases in Brazil

Diversity of phlebotomine sand flies and molecular detection of trypanosomatids species in Brumadinho, Minas Gerais, Brazil

Aline Tanure, Felipe Dutra Rêgo, Gabriel Barbosa Tonelli, Aldenise Martins Campos, Paloma Helena Fernandes Shimabukuro, Célia Maria Ferreira Gontijo, Gustavo Fontes Paz, José Dilermando Andrade-Filho*

Grupo de Estudos em Leishmanioses – Instituto René Rachou – FIOCRUZ Minas – Av. Augusto de Lima, 1715 Barro Preto, CEP 30190–002, Belo Horizonte, Minas Gerais, Brasil

*corresponding author

E-mail: jose.andrade@fiocruz.br

Abstract

This study aimed to investigate the phlebotomine sand fly fauna and perform the molecular detection of trypanosomatids in sand flies collected in the Casa Branca district of Brumadinho municipality, located in the metropolitan region of Belo Horizonte, state of Minas Gerais, Brazil, an endemic area of visceral (VL) and tegumentary leishmaniases (TL). Samples were collected bimonthly from May 2013 to July 2014 using two HP light traps exposed in nine peridomiciliary areas with previous reports of VL or TL (total 18 traps) for three consecutive nights. DNA was extracted from female sand flies and subjected to ITS1-PCR. Subsequently DNA sequencing was conducted on PCR products for species level identification. A total of 23 species were found among 16,771 sand flies collected. The most abundant species was *Nyssomyia whitmani* (Antunes & Coutinho, 1939) (70.9%), followed by *Lutzomyia longipalpis* (Lutz & Neiva, 1912) (15.2%) and *Migonemyia migonei* (França, 1920) (9.1%). A total of 4,913 females were analyzed individually or in pools of 1-20 specimens for the molecular detection of *Leishmania* parasites. The DNA of *Leishmania amazonensis* was detected in four pools, belonging to *Nyssomyia whitmani*; DNA of *Le*. *braziliensis* was detected in one pool of *Psychodopygus lloydi*, and *Leishmania* sp. was detected pools of *Nyssomyia whitmani* (7) and *Lutzomyia longipalpis* (1) In addition, the DNA of *Herpetomonas* sp. and *Crithidia* sp. was detected in female sand flies and identified using ITS1-PCR. Our results suggest that *Ny*. *whitmani* is a vector in the study area. In addition, the molecular detection of *Le*. *amazonensis* suggests the presence of this species in a sylvatic cycle between vertebrate and invertebrate hosts in the region of Casa Branca. Our data also reveal the occurrence of other non-*Leishmania* trypanosomatids in sand flies in Casa Branca District.

Key words: Leishmaniases; *Leishmania braziliensis*; *Leishmania amazonensis*; *Lutzomyia longipalpis*; *Nyssomyia whitmani*; Trypanosomatidae

Introduction

Phlebotomine sand flies (Diptera: Psychodidae: Phlebotominae) are known to be natural hosts of etiological agents such as bacteria, viruses and protozoa [1, 2]. Among these agents, are species of the genus *Leishmania,* which are transmitted during blood feeding by female sand flies [3]. In the New World, some genera of sand flies (*Lutzomyia*, *Psychodopygus*, *Pintomyia*, *Nyssomyia*, among others) are involved in the transmission of these parasites to vertebrate hosts [4].

More than 1,000 species of phlebotomine sand flies are known throughout the world, of which about of 530 species are in the Neotropical Region and Brazil has a greater diversity of species of phlebotomine sand flies than any other nation in the world, with approximately 274 species recorded [5].

In Brazil, some species of sand flies are adapted to urban and peri-urban environments, and exhibit a high degree of anthropophily. The species *Lutzomyia longipalpis* is incriminated as the main vector of *Leishmania infantum*, the etiologic agent of visceral leishmaniasis (VL) [6, 7]. Other species of phlebotomine sand flies are important in the transmission of *Leishmania braziliensis,* which causes tegumentary leishmaniasis (TL), such as *Nyssomyia whitmani* and *Nyssomyia intermedia* in the southeast region of Brazil [8, 9].

Other trypanosomatids such as *Endotrypanum*, *Herpetomonas*, *Crithidia*, *Blastocrithidia*, *Leptomonas*, and *Sauroleishmania* are likely to be found in the digestive tract of sand flies, since they may be share the same habitat [~~1, ~~10, 11]. Several authors have reported the presence of non-*Leishmania* trypanosomatids DNA in

sand flies by means of molecular techniques [12, 13, 14, 15]. However, the identification of these protozoa has not been considered relevant from the epidemiological point of view. In a recent phylogenetic study, *Leishmania colombiensis*, an important species associated with human cases of TL in South America, was classified in the genus *Endotrypanum* suggesting that the non-*Leishmania* trypanosomatids may also cause leishmaniases and therefore, studies are needed to better explain the role of the sand flies in the transmission of these parasites to humans [16].

Intense urbanization over the last decades has caused changes in natural habitats, especially those occupied by phlebotomine sand flies. Some species are able to withstand several environmental modifications that may occur in natural breeding sites and successfully adapt to urban environments [17, 18]. In some regions, the presence of vector species in the human environment may be associated with the emergence of autochthonous cases of leishmaniases, as occurs in the state of Minas Gerais, where cases have been recorded in several regions [19, 20, 21, 22] including the metropolitan region of Belo Horizonte, Minas Gerais state capital [23, 24]. In this context, the municipality of Brumadinho (Minas Gerais) has reported an increasing number of cases of leishmaniases over the last years. In the period of 2007 to 2014, 28 cases of TL and 13 cases of VL were recorded in the municipality. The district of Casa Branca, which belongs to the municipality of Brumadinho, is responsible for many of these cases, including three TL and four VL cases (Source: Municipal Secretary of Health of Brumadinho, MG). In this area, Pereira et al. [25] recorded the presence of *Leishmania* spp. in rodents and marsupials. Given this situation, this study aimed to describe the sand fly fauna, detect and identify by molecular assay the presence of trypanosomatids in female sand flies from Casa Branca.

Material and Methods

Study area

The municipality of Brumadinho (20º08'36" S and 44º11'59" W) is located within the metropolitan region of Belo Horizonte, approximately 50 km from the state capital. It covers an area of 639,434 km² with a total population of 38,373 inhabitants [26]. The municipality is located in the humid subtropical zone with dry winter and hot summer (Cwa – Köppen's climate classification) situated in an area that includes the "Quadrilátero Ferrífero", an area rich in ferruginous fields, with industrial mining as the primary economic activity.

The study was conducted in the district of Casa Branca (20°6'2.58"S and 44°2'59.45" W), located in the northern part of the municipality of Brumadinho (Figure 1). Casa Branca is also bordered by the Parque Estadual Serra do Rola Moça conservation unit, the third largest urban park in Brazil, which is located in the transition zone between Atlantic Forest and Cerrado and has abundant natural resources. About eight kilometers west of Casa Branca is a village called Córrego do Feijão, where, in January 2019, an ore tailing dam ruptured, causing great environmental and social impact in all areas of Brumadinho municipality, as well as the death of 270 people.

Fig 1. Location of the study area in the district of Casa Branca, Brumadinho, Minas Gerais, Brazil.

Sand fly collection

Sand flies were collected bimonthly between May 2013 and July 2014, using two CDC light traps (model HP) [27], which were exposed in peridomicile areas of nine houses (total 18 traps). The traps were exposed at 5 PM and collected after three consecutive nights at 8 AM. Collection sites with previous report of human or canine leishmaniases cases were selected. The owners of the residences where the sand flies were collected authorized verbally the installation of the traps during the period of study

Collected sand flies were taken to the laboratory where they were killed in ether, screened and clarified. The males and the females of the collections of July/2013, November/2013, March/2014 and July/2014 were mounted in Canada balsam on a glass slide for species identification. The females from the collections of May/2013, September/2013, January/2014 and May/2014 were dissected by removing the last three segments of the abdomen and head, mounted on a glass slide in Berlese liquid. The remainder of the body was individually conditioned in a sterile 1.5 mL tube for later assembly of the pools for DNA extraction.

Species identification was determined using the classification proposed by Galati [28]. The females of *Evandromyia sallesi* and *Evandromyia cortelezzii*, were identified as belonging to the cortelezzii complex because they are undistinguishable by their morphology [29]. The abbreviation of the genera followed the proposal of Marcondes [30]

Climate data

The climate data of average monthly temperature, relative humidity and rainfall, were obtained from the Automatic Weather Station of Ibirité located in the Parque Estadual Serra do Rola Moça and approximately 10 km from the study site in Casa Branca.

Statistical analyses

To evaluate species abundance the index of species abundance "ISA", and the standardized index of species abundance "SISA" were calculated [31]. The values of SISA range from 0 to 1, with values closer to 1 representing the most abundant species. The Shannon index (H) and evenness index (J) [32] were used to determine species diversity and evenness of species abundance, respectively. To assess relationships between sand fly abundance and the climate variables at Casa Branca during the study period, the Spearman correlation test was used. The DNA detection rate was calculated considering one phlebotomine sand fly infected per positive pool.

DNA extraction and identification of *Leishmania*

Females without presence of blood in their abdomen were individually stored dry at -20 °C in a 1.5 mL tube or grouped into pools of up to 20 individuals of the same species, date and site of collection. Females were subjected to DNA extraction using the Gentra Puregene® kit (Qiagen, Valencia, CA, USA) following the manufacturer's protocol.

After DNA extraction, the Polymerase Chain Reaction technique directed to the internal transcribed spacer 1 (ITS1-PCR) of *Leishmania* ribosomal DNA was performed, which amplifies a region of 300-350 bp, using the pair of primers LITSR: 5' CTGGATCATTTTCCGATG 3 'and L5.8S: 5'-TGATACCACTTATCGCACTT 3' [33, 34].

In all reaction set-ups, negative controls containing only the PCR-reagents and replacing the DNA by nuclease free water and positive controls were included: *Leishmania amazonensis* (IFLA/BR/67/PH8), *Le*. *braziliensis* (MHOM/BR/75/M2903), *Le*. *infantum* (MHOM/BR/74/PP75) and *Le*. *guyanensis* (MHOM/BR/75/M4147). As

sand flies may host other parasites, exogenous controls have been added in PCR reactions: *Crithidia fasciculata* (FIOCRUZ-COLPROT 048), *Endotrypanum monterogeii* (FIOCRUZ-COLPROT 151), *Herpetomonas samuelpessoai* (FIOCRUZ-COLPROT 067), *Leptomonas collosoma* (FIOCRUZ-COLPROT 073) and *Phytomonas serpens* (FIOCRUZ-COLPROT 189). These controls were kindly provided by the Coleção de Protozoários of the Laboratório de Biologia Molecular e Doenças Endêmicas (FIOCRUZ-COLPROT) of the Instituto Oswaldo Cruz, Rio de Janeiro, Brazil).

The amplicons were visualized in a 2% agarose gel stained with ethidium bromide (10mg/mL) with a 100 bp DNA Step Ladder provided as molecular length size standard. The PCR-positive products were subjected to the PCR-RFLP technique using the *Hae*III digestion enzyme as described by Schönian et al. [34].

For confirmation, all PCR amplicons were sequenced. The PCR-products were purified using the QIAquick® PCR Purification Kit (Qiagen, Valencia, CA, USA) as described by the manufacturer. The sequencing reactions were prepared in duplicate using 1 μL of the purified product, 1 μL of primer at 5 pmol (antisense and sense in separate tubes), 1 μL Big Dye Terminator v3.1 Cycle Sequencing Kit (Applied Biosystems, Foster City, CA, USA) and distilled water to a final volume of 10 μL. Sequences were then generated by the ABI 3730x1 DNA Analyzer and Geneious (v. 9.1.3) was used to check the electropherograms, align sequences and compare with the sequences deposited in the GenBank database through the BLAST alignment algorithm, considering the two most similar organisms according to identity above 90% for genus level and 98% for species level. These sequences are available in Genbank (Accession numbers MN638721-MN638737).

Ethics

The collections were carried out with the due permission of the Brazilian environmental agencies (SISBIO number 15237-2). The sand flies were deposited in the Coleção de Flebotomíneos of the Instituto René Rachou/Fiocruz (FIOCRUZ/COLFLEB).

Results

Sand fly fauna

During the study period, 16,771 sand flies of 23 species and eight genera were collected in Casa Branca: *Brumptomyia* sp., *Evandromyia cortelezzii*, *Ev. edwardsi*, *Ev. evandroi*, *Ev. lenti*, *Ev. sallesi*, *Ev. teratodes*, *Ev. termitophila*, *Ev. tupynambai*, *Lutzomyia amarali*, *Lu. ischyracantha*, *Lu. longipalpis*, *Migonemyia migonei*, *Nyssomyia intermedia*, *Ny. whitmani*, *Pintomyia bianchigalatiae*, *Pi. fischeri*, *Pi. mamedei*, *Pi. misionensis*, *Pi. monticola*, *Pi. pessoai*, *Psathyromyia pascalei*, and *Psychodopygus lloydi*.

The most abundant species was *Ny. whitmani* (70.9%; SISA 0.74), followed by *Lu. longipalpis* (15.2%; SISA 0.53) and *Mg. migonei* (9.1%; SISA 0.51) (Table 1) (Figure 2). The Shannon (H) and evenness (J) indexes were low for the study area (H’= 0.9946229; J’= 0.308997).

Fig 2. Standardized index of species abundance of species collected at Casa Branca, Brumadinho, Minas Gerais in May 2013 to July 2014.

Among the genera, *Evandromyia* was the richest with eight species, followed by *Pintomyia* with six (Table 1). The month of May 2013 showed the greatest abundance of species collected (7,054 sand flies) while July 2014 had the lowest (266). The highest species richness was observed in May 2014 with a total of 18 species (Table 1).

The climate parameters of temperature and relative humidity presented short variation during the study period. Rainfall ranged from periods of no rain in July 2013 to a maximum of 128.2 mm³ in January 2014. However, there was little difference in precipitation levels during the other months of the study (Figure 3). There is a trend of a positive correlation between the number of individuals and temperature (0.19 - Spearman correlation; p-value > 0.05). There was no correlation between the humidity and rainfall with the abundance of sand flies during the study (0.012 and -0.024 Spearman correlation, respectively; p-value > 0.05). There was no significant correlation between the number of sand flies collected each month and temperature, relative humidity or rainfall (p-value > 0.05 – Spearman correlation).

Fig 3. The relationship between seasonal variation in the number of individuals of species of sand flies collected and the climate variables at Casa Branca, Brumadinho, Minas Gerais in May 2013 to July 2014.

Molecular studies

Of a total of 4,913 females, 47 were individually tested and 4,866 grouped in 311 pools. Table 2 presents information about the species collected and their distributions for molecular analysis. *Nyssomyia whitmani* was the most abundant and therefore yielded the largest number of pools (4,128 specimens/220 pools), followed by *Lu. longipalpis* (248/19), *Mg. migonei* (144/14) and *Ps. lloydi* (115/18).

No sample individually analyzed in this study was PCR positive for *Leishmania*. A total of 13 pools were PCR-positive for *Leishmania*, being one pool of *Lu. longipalpis*, 11 of *Ny. whitmani* and one of *Ps. lloydi* (Table 3). The infection rate obtained for *Leishmania* was 0.26% (13/4,913). Twelve pools showed high similarity

values when compared to Genbank sequences (>98%), allowing us to confirm sample identity either to species or genus level; one pool of *Lu. longipalpis* presented a low similarity level (80%) to publicly available *Leishmania* sequences due to low quality of the sequences, which contained a high number of ambiguous sites.

A total of four pools of *Ny. whitmani* were positive for *Le. amazonensis* (0.08%) but it was not possible get to species level in other seven pools of this sand flies species, therefore, sequences were left as undetermined (*Leishmania* sp.) (Table 3). One pool of the species *Ps. lloydi* was positive for DNA of *Le. braziliensis* (0.02%). *Lutzomyia longipalpis* was positive for DNA of *Leishmania* sp. (0.02%) (Table 3).

Positive pools (n = 5) with a natural infection rate of 0.10% (5/4,913) for ITS1-PCR revealed the presence of bands of inconsistent size (400-500 bp) with fragments for *Leishmania* (~350 bp). These results led us to investigate the identity of these fragments and three trypanosomatid species were identified from the ITS1 fragments (Table 3).

One pool of *Ny. whitmani* and of cortelezzii complex were positive for *Herpetomonas* sp. One pool of *Ps. lloydi* and two pools of *Ny. whitmani* were positive for *Crithidia* sp. (Table 3). Sequence similarity to Genbank sequences ranged between 90-97%.

Discussion

The diversity of species found in this study may be explained by the fact that Casa Branca is located next to Parque Estadual Serra do Rola Moça, and so the residences selected for sampling are close to forest environments that are rich in natural resources. At the same time, the peridomicile areas of these households were conducive to the breeding and maintenance of sand fly populations including the presence of vegetation (fruit trees), the accumulation of organic matter on the ground and the

housing of domestic animals (chickens, dogs, pigs), which can serve as sources of food and shelter for sand flies.

In the present work, *Ny. whitmani* was the most abundant sand fly in the samples peridomicile areas (70.85%), being found in high density close to residences. This species has frequently been found in areas endemic for TL in southeastern Brazil, in the states of Espírito Santo, Minas Gerais and São Paulo, and is incriminated as a major vector of *Le. braziliensis* in some regions of Brazil [35, 36, 37, 38]. Furthermore, *Ny. whitmani* has also been found positive for DNA from *Le. infantum* in the state of Minas Gerais [39, 40], although nothing is known about this species role in the transmission of this species of *Leishmania*.

Lutzomyia longipalpis, the main vector of *Le. infantum* in Brazil, was the second most abundant species in Casa Branca (15.22%), and was also found to be frequent near houses. This finding shows the adaptation of this species to an environment undergoing development, which are the conditions often found among the residences of Casa Branca where dogs, chickens and pigs are being bred, and also confirm the high degree of adaptation to the anthropic environment of this species.[41, 42].

Migonemyia migonei was the third most captured species in the study area with 9.14%, and is also considered an anthropophilic species [43, 44]. In a study conducted in the state of Pernambuco, *Mg. migonei* predominantly in peridomiciliary areas and this species has been naturally infected by *Le. infantum*, suggesting that *Mg. migonei* could be serving as the vector species in endemic areas for visceral leishmaniasis where *Lu. longipalpis* is absent [45].

The genus *Evandromyia* presented the highest number of species (8). Although there are no proven vector species in this genus, natural infections or *Leishmania* DNA has been detected in individuals of some of its species. In studies conducted in the state

Formatted: Indent: First line: 0.49"

of Minas Gerais, Carvalho et al. [46] detected DNA of *Le. infantum* in *Ev. cortelezzii* and Saraiva et al. [47] found female of *Ev. sallesi* naturally infected by the same species of *Leishamania*. These findings indicate that the species of the cortelezzii complex (females of *Ev. cortelezzii* and *Ev. sallesi* do not differ morphologically) hold some epidemiological significance because they have been found with some frequency in the urban environment [48].

The genus *Pintomyia* was the second richest, with six species. Some species of this genus have been shown to frequent peridomicile areas in association with domestic animal shelters, and it is known to be anthropophilic [43]. In addition, some species have been found with *Leishmania* DNA in Brazil, thus being associated with transmission of the disease in endemic areas [49, 50, 51].

The highest standardized index of species abundance (SISA) in this study was found for *Ny. whitmani* (0.74). Regarding diversity and evenness, despite the study area being quite diverse (H '= 0.995), the abundances of the species were not similar, with most individuals belonging to the dominant species *Ny. whitmani*. This likely explains the low value for the evenness index (J '= 0.309), since the great abundance of one species tends to cause less uniformity among the others.

The correlations detected between seasonal variation in sand fly abundance and climate data (temperature, humidity and rainfall) did not reach statistical significance according to the Spearman correlation test. This can be explained by the small amount of variation in relative humidity, temperature and rainfall of each month during the study period, and a greater amount of variation may be needed to influence the population dynamics of sand flies. Furthermore, other factors not tested could be responsible for the variation in the number of sand flies collected each month, such as changes at sites throughout the year such as the presence of hosts, the type of

vegetation, cleanliness of sites or even microclimate characteristics. All of these factors could influence the sand fly populations throughout the year in Casa Branca.

Another important factor is the study period. It is widely known that to ensure greater reliability of seasonal data it is desirable that a study encompass at least two consecutive years so that they can be compared and reduce the error caused by atypical climate conditions that may be occur throughout the year. So the number of bimonthly collections may not have been sufficient to ascertain the veracity the relationships of temperature, humidity and rainfall with the number of individual sand flies collected.

With regard to the feeding habit of sand fly species found in the Casa Branca District, it is interesting to highlight the study carried out in the same area by Carvalho et al. [52] who showed that several of them are eclectic about eating habits. *Nyssomyia whitmani* and *Mg. migonei* fed more frequently on birds, followed by dogs and *Lu. longipalpis* in humans followed by dogs.

Nyssomyia whitmani-pools were positive for *Le. amazonensis*. Changes in the natural environment may have triggered a new epidemiological profile of leishmaniases that could involve *Leishmania amazonensis*, an alternative vector (i.e. *Ny. whitmani*) and new reservoirs (domestic and wild). Similar fact has been reported in the literature for other species of sand flies [53, 54]. Added to this, in an experimental infection study conducted by Fonteles et al. [55] it was possible to infect *Ny. whitmani* with *Le. amazonensis*, showing that this sand fly species has the potential to become infected by other *Leishmania* species, being considered as a probable vector of *Le. amazonensis* in the state of Maranhão. In view of this, *Ny. whitmani* could be act in the transmission cycle of *Le. amazonensis* in Casa Branca.

The species *Ps. lloydi* has been associated with the transmission of *Le. braziliensis* in wild areas of Minas Gerais [56, 57] and was also found in this state with DNA of *Le.*

infantum [58]. *Psychodopygus lloydi* was positive for DNA of *Leishmania braziliensis*, suggesting that this sand fly could maintain a wild cycle of TL in the District of Casa Branca.

We found three species of sandflies with DNA from trypanosomatids other than *Leishmania*: *Ny. whitmani* with *Herpetomonas* sp. (two pools) and *Crithidia* sp. (one pool); *Ps. lloydi* with a pool of *Crithidia* sp. and a pool of the cortelezzii complex with Herpetomonas sp. This record is important, since recently other trypanosomatids have been found in humans [59, 60]

Only the PCR-RFLP technique using the restriction enzyme *Hae III* did not prove to be a good tool to differentiate the species of the *Leishmania* genus from other trypanosomatids detected in the pools of sand flies females in this paper. However, when associated with sequencing, the technique proved to be effective for specific identification of trypanosomatids. This fact can be explained by the molecular target used (ITS1) that does not distinguish in a same pool *Leishmania* species from other trypanosomatids, generating overlapping restriction profiles that made it difficult to identify the species, since the PCR-RFLP technique was initially described to generate restriction profiles for species of the genus *Leishmania* in clinical samples of patients, and not for the other trypanosomatids [34]. Thus, the use of this technique did not produce a satisfactory result when used for characterization of other trypanosomatids (non-*Leishmania*) detected in sand flies.

Currently, molecular techniques for the detection of trypanosomatids are being widely used. The combination of molecular techniques allows us to determine the species of the trypanosomatid. The use of the PCR technique directed to the mini-circle of kDNA demonstrated the presence of *Herpetomonas samuelpessoai* in *Pintomyia fischeri* in the State of Espírito Santo [50], and in a study carried out in Brasília using

different molecular markers, trypanosomatid DNA from the genus *Blastocrithidia* was found for the first time in *Ny*. *whitmani* and *Trypanosoma* sp. in *Evandromyia evandroi* [14]. The use of the ITS1 target allowed the detection of *Crithidia fasciculata* in sand flies in Cerrado area in the state of Minas Gerais [15]. Although these findings demonstrate the presence of trypanosomatid DNA in sand flies in Brazil, further studies are needed to clarify the associations of sand flies with trypanosomatid species.

DNA sequencing has generated good results when used for detection of *Leishmania* in sand flies [22, 61]. However, there are limitations to the use of ITS1 for *Leishmania* detection in sand flies, such as: i. the high number of copies of ITS1 yields poor quality sequencing reads; ii. ITS1 is highly conserved and has been amplifying fragments of other trypanosomatid species in field samples; iii. lower taxonomic resolution for species such as *Le. braziliensis* and *Le. guyanensis*. Thus, the final result depends on the quality of the sample, along with molecular biology techniques (DNA extraction, PCR, sequencing and phylogenetic analyzes). Despite these limitations, our results allow the identification of other species of trypanosomatids in addition to the *Leishmania* genus in sand flies.

Conclusion

This is the first study of a sand fly fauna in the municipality of Brumadinho, Minas Gerais. Our findings show that the Casa Branca locality has a diverse sand fly fauna with species that have been previously reported in the state of Minas Gerais. The *Ny*. *whitmani* species is the probable vector of *Le*. *braziliensis* in Casa Branca, and may also be involved in the transmission of *Le*. *amazonensis*.

The knowledge of the interactions between sand flies and the species of *Leishmania* and other trypanosomatids detected in this study shows that the infection may be occurring in the peridomiciliary environment in the study area. In addition,

these results help to understand the dynamics of the leishmaniases transmission cycle in Casa Branca providing support for disease control actions in the region. The large number of pools of infected sand flies raises an issue that has been neglected and is of great importance, which would be the circulation of these parasites among phlebotomine insects, and that some molecular techniques can detect the presence of these parasites in sand flies, requiring attention to interpretation results.

Acknowledgments: We thank Surveillance System in Health Brumadinho for providing epidemiological data and we thank the residents of the collection sites for making the completion of the work possible.

References

1. Alvar J, Vélez ID, Bern C, Herrero M, Desjeux P, et al. Leishmaniasis Worldwide and Global Estimates of Its Incidence. PLoS ONE 2012; 7(5): e35671.~~Shaw J, Rosa AT, Souza A, Cruz AC. Transmissão de outros agentes: os flebotomíneos brasileiros como hospedeiros e vetores de determinadas espécies. In Rangel EF, Lainson R, editors. Flebotomíneos do Brasil: Rio de Janeiro: Fundação Oswaldo Cruz. 2003. pp. 337-351.~~
2. Alkan C, Moin Vaziri V, Ayhan N, Badakhshan M, Bichaud L, Rahbarian N, et al. Isolation and sequencing of Dashli virus, a novel Sicilian-like virus in sandflies from Iran; genetic and phylogenetic evidence for the creation of one novel species within the *Phlebovirus* genus in the *Phenuiviridae* family. PLoS Negl Trop Dis. 2017; 11: e0005978.

3. Lestinova T, Vlkova M, Votypka J, Volf P, Rohousova I. *Phlebotomus papatasi* exposure cross-protects mice against *Leishmania major* co-inoculated with *Phlebotomus duboscqi* salivary gland homogenate. Acta Trop. 2015; 144: 9–18.~~Deane MP, Deane LM. Infecção natural do *Phlebotomus longipalpis* por leptomonas, provavelmente de *Leishmania donovani*, em um foco de calazar, no ceara. O Hospital. 1954; 6: 697-702.~~

4. Brazil RP, Rodrigues AAF, Andrade-Filho JD. Sand Fly Vectors of Leishmania in the Americas - A Mini Review. Entomol Ornithol Herpetol Curr Res. OMICS International.~~;~~ 2014; 4: 144.

5. Shimabukuro PHF, Andrade AJ, Galati EAB. Phlebotominae in Taxonomic Catalog of the Brazilian Fauna.~~Catálogo Taxonômico da Fauna do Brasil.~~ PNUD. 2018. Available from: http://fauna.jbrj.gov.br/fauna/faunadobrasil/3297.

6. Deane LM, Deane MP. Visceral leishmaniasis in Brazil: geographical distribution and transmission. Rev Inst Med Trop São Paulo. 1962; 4: 198-212.

7. Lainson R, Shaw JJ. The role of animals in the epidemiology of South American leishmaniasis. In: Lumsden EDA, editor. Biology of the Kinetoplastida. London and New York: Academic Press. 1979; 2: 1-116.

8. Mayrink W, Williams P, Coelho M V, Dias M, Martins AV, Magalhães PA, et al. Epidemiology of dermal leishmaniasis in the Rio Doce Valley, State of Minas Gerais, Brazil. Ann Trop Med Parasitol. 1979; 73: 123-137.

9. Andrade Filho JD, Galati EAB, Falcão AL. *Nyssomyia intermedia* (Lutz & Neiva, 1912) and *Nyssomyia neivai* (Pinto, 1926) (Diptera: Psychodidae: Phlebotominae) geographical distribution and epidemiological importance. Mem Inst Oswaldo Cruz. Fundação Oswaldo Cruz; 2007; 102: 481-487.

Formatted: Portuguese (Brazil)

Formatted: Portuguese (Brazil)

Formatted: Portuguese (Brazil)

Formatted: Portuguese (Brazil)

Formatted: Portuguese (Brazil)

Formatted: English (United States)

Formatted: Font: Not Bold

Formatted: English (United States)

Formatted: Portuguese (Brazil)

10. Christensen HA, Herrer A. Neotropical Sand Flies (Diptera: Psychodidae), Invertebrate Hosts of *Endotrypanum Schaudinni* (Kinetoplastida: Trypanosomatidae). J Med Entomol. 1976; *13*: 299-303.Pinheiro FG, Luz SLB, Franco AMR. Infecção natural por tripanosomatídeos (Kinetoplastida: Trypanosomatidae) em *Lutzomyia umbratilis* (Diptera: Psychodidae) em áreas de leishmaniose tegumentar americana no Amazonas, Brasil. Acta Amaz. 2008; 38: 165-172.

11. Kalantari M, Motazedian MH, , Soltani Z, Soltani A, Azizi K. Bionomics of phlebotomine sand flies species (Diptera: Psychodidae) and their natural infection with *Leishmania* and *Crithidia* in Fars province, southern Iran Mohsen. J Parasit Dis. 2018; 42: 511-518

Gomes, LHM, Franco AMR. Variação mensal e infecção natural em *Lutzomyia umbratilis* Ward & Fraiha, 1977, *Lutzomyia anduzei* Rozeboom, 1942, *Lutzomyia flaviscutellata* Mangabeira, 1942 e *Lutzomyia olmeca nociva* Young & Arias, 1982 (Diptera: Psychodidae) por tripanosomatídeos (Kinetoplastida: Trypanosomatidae) em área de treinamento militar na Amazônia, Amazonas, Brasil. Rev Pan-Amaz Saude. 2011; 2: 75-76.

12. Kato H, Uezato H, Sato H, Bhutto AM, Soomro FR, Baloch JH, et al. Natural infection of the sand fly *Phlebotomus kazeruni* by Trypanosoma species in Pakistan. Parasites & Vectors. 2010; 3: 10.

13. Nzelu CO, Kato H, Puplampu N, Desewu K, Odoom S, Wilson MD, et al. First Detection of *Leishmania tropica* DNA and *Trypanosoma* Species in *Sergentomyia* Sand Flies (Diptera: Psychodidae) from an Outbreak Area of Cutaneous Leishmaniasis in Ghana. PLoS Negl Trop Dis. 2014; 8: 2630.

Formatted: Font color: Auto
Formatted: Font color: Auto
Formatted: Font color: Auto
Field Code Changed
Formatted: Font color: Auto
Field Code Changed
Formatted: Font color: Auto
Field Code Changed
Field Code Changed
Field Code Changed

14. Ferreira TS, Minuzzi-Souza TTC, Andrade AJ, Coelho TO, Rocha DA, Obara MT, et al. Molecular detection of *Trypanosoma* sp. and *Blastocrithidia* sp. (Trypanosomatidae) in phlebotominesand flies (Psychodidae) in the Federal District of Brazil. Rev Soc Bras Med Trop. 2015; 48: 776-779.

15. Saraiva L, Reis AS, Rugani JMN, Pereira AAS, Rêgo FD, Lima ACVMR, et al. Survey of sand flies (Diptera: Psychodidae) in an environmentally protected area in Brazil. Plos One. 2015; 10: 1-5.

16. Espinosa OA, Serrano MG, Camargo EP, Teixeira MM, Shaw JJ. An appraisal of the taxonomy and nomenclature of trypanosomatids presently classified as *Leishmania* and *Endotrypanum*. Parasitology. 2016; 15 :1-13.

17. Harhay MO, Olliaro PL, Costa DL, Costa CHN. Urban parasitology: visceral leishmaniasis in Brazil. Trends Parasitol. 2011; 27: 403-409.

18. Silva TAM, Coura-Vital W, Barbosa DS, Oiko CSF, Morais MHF, Tourinho BD, et al. Spatial and temporal trends of visceral leishmaniasis by mesoregion in a southeastern state of Brazil, 2002-2013. PLoS Negl Trop Dis. 2017; 11: e0005950.

19. Gontijo CMF, Silva ES, Fuccio MB, Sousa MCA, Pacheco RS, Dias ES, et al. Epidemiological studies of an outbreak of cutaneous leishmaniasis in the Rio Jequitinhonha Valley, Minas Gerais, Brazil. Acta Trop. 2002; 81: 143-150.

20. Prado PF, Rocha MF, Sousa JF, Caldeira DI, Paz GF, Dias ES. Epidemiological aspects of human and canine visceral leishmaniasis in Montes Claros, State of Minas Gerais, Brazil, between 2007 and 2009. Rev Soc Bras Med Trop. 2011; 44: 561-566.

21. Barata RA, Peixoto JC, Tanure A, Gomes ME, Apolinário EC, Bodevan EC, et al. Epidemiology of visceral leishmaniasis in a reemerging focus of intense

transmission in Minas Gerais State, Brazil. Biomed Res Int. 2013; 2013 Article ID 405083

22. Saraiva L, Leite CG, Lima AC, Carvalho LO, Pereira AA, Rugani JM, et al. Seasonality of sand flies (Diptera: Psychodidae) and *Leishmania* DNA detection in vector species in an area with endemic visceral leishmaniasis. Mem Inst Oswaldo Cruz. 2017; 112: 309-318.

23. Saraiva L, Andrade Filho JD, Falcão AL, de Carvalho DAA, de Souza CM, Freitas CR, et al. Phlebotominae fauna (Diptera: Psychodidae) in an urban district of Belo Horizonte, Brazil, endemic for visceral leishmaniasis: Characterization of favored locations as determined by spatial analysis. Acta Trop. 2011; 117: 137-145.

24. Souza CM, Pessanha JE, Barata RA, Monteiro EM, Costa DC, Dias ES. Study on Phlebotomine Sand Fly (Diptera: Psychodidae) Fauna in Belo Horizonte, State of Minas Gerais, Brazil. Mem Inst Oswaldo Cruz. 2004; 99: 795-803.

Morais MHF, Fiuza V de OP, Araújo VEM de, Menezes FC de, Carneiro M. Avaliação das atividades de controle da leishmaniose visceral em Belo Horizonte, Minas Gerais, 2006-2011. Epidemiol e Serviços Saúde. Secretaria de Vigilância em Saúde - Ministério da Saúde do Brasil; 2015; 24: 485–496.

25. Pereira AAS, Ferreira EC, Lima ACVMR, Tonelli GB, Rêgo FD, Paglia AP, et al. (2017) Detection of *Leishmania* spp in silvatic mammals and isolation of *Leishmania*(*Viannia*) *braziliensis* from *Rattus rattus* in an endemic area for leishmaniasis in Minas Gerais State, Brazil. PLoS ONE. 2017; 12: e0187704.

26. IBGE. Estimativa da População, Brumadinho, Minas Gerais, Brasil. Instituto Brasileiro de Geografia e Estatística (IBGE). 2016.

Formatted: Portuguese (Brazil)

http://www.cidades.ibge.gov.br/xtras/perfil.php?lang=&codmun=310900. Accessed 20 Jul 2016.

27. Pugedo H, Barata RA, França-Silva JC, Silva JC, Dias ES. HP: um modelo aprimorado de armadilha luminosa de sucção para a captura de pequenos insetos. Rev Soc Bras Med Trop. 2005; 38: 70-72.

28. Galati EAB. Phlebotominae (Diptera, Psychodidae): Classification, morphology, and terminology of adults and identification of American taxa In: Rangel E.F. & Shaw J.J. (eds.) Brazilian sand flies: Biology, taxonomy, medical importance and control. Rio de Janeiro, Brazilian Ministry of Health. Oswaldo Cruz Foundation, 2018, pp.9–212.

~~Galati EAB. Morfologia e Taxonomia: Morfologia, terminologia de adultos e identificação dos táxons da América. In Rangel EF, Lainson R, editors. Flebotomíneos do Brasil: Rio de Janeiro: Fundação Oswaldo Cruz. 2003. pp. 53-175.~~

29. Szelag ESA, Rosa JR, Galati EAB, Andrade Filho JD, Salomón OD.Considerations on the species complex of the Cortelezzii series (Diptera: Psychodidae) and description of *Evandromyia chacuensis* sp. nov., a new phlebotomine species of the Chaco Region, Argentina. J Med Entomol. 2018; 55: 902–909.

~~Carvalho GML, Brazil RP, Falcão AL, Andrade Filho JD. Distribuição geográfica do Complexo *cortelezzii* (Diptera: Psychodidae: Phlebotominae) no Brasil. Neotrop Entomol. 2009; 38: 876-879.~~

30. Marcondes CB. A proposal of generic and subgeneric abbreviations for phlebotomine sandflies (Diptera: Psychodidae: Phlebotominae) of the world. Entomol News. 2007; 118: 351-356.

Formatted: English (United States)

Formatted: English (United States)

Formatted: English (United States)

31. Roberts DR, Hsi BP. An Index of Species Abundance for Use with Mosquito Surveillance Data. Environ Entomol. 1979; 8: 1007-1013.

32. Hayek LAC, Buzas MA. Surveying Natural Populations. New York, Columbia University Press. 1997: 347-389.

33. El Tai N O, Osman OF, El Fari M, Presber W, Schönian G. Genetic heterogeneity of ribosomal internal transcribed spacer (its) in clinical samples of *Leishmania donovani* spotted on filter paper as revealed by single-strand conformation polymorphisms (sscp) and sequencing. Trans Royal Soc Trop Med Hyg. 2000; 94: 1-5.

34. Schönian G, Nasereddin A, Dinse N, Schweynoch C, Schallig HD, Presber W et al. PCR diagnosisand characterization of *Leishmania* in local and imported clinical samples. Diagn Microbiol Infect Dis. 2003; 47: 349-358.

35. Colla-Jacques FE, Casanova C, Prado AP. Study of sand fly fauna in an endemic area of American cutaneous leishmaniasis and canine visceral leishmaniasis in the municipality of Espírito Santo do Pinhal, São Paulo, Brazil. Mem Inst Oswaldo Cruz. 2010; 105): 208-215. ~~Camargo-Neves VLF, Gomes AC, Antunes JLF. Correlação da presença de espécies de flebotomíneos (Diptera: Psychodidae) com registros de casos da leishmaniose tegumentar americana no Estado de São Paulo, Brasil. Rev Soc Bras Med Trop. 2002; 35: 299-306.~~

36. Costa SM da, Cechinel M, Bandeira V, Zannuncio JC, Lainson R, Rangel EF. *Lutzomyia* (*Nyssomyia*) *whitmani* s.l . (Antunes & Coutinho, 1939) (Diptera: Psychodidae: Phlebotominae): geographical distribution and the epidemiology of American cutaneous leishmaniasis in Brazil Mini-review. Mem Inst Oswaldo Cruz. Fundação Oswaldo Cruz. 2007; 102: 149-153.

37. Virgens TM, Santos CB, Pinto IS, Silva KS, Leal FC, Falqueto A. Phlebotomine sand flies (Diptera, Psychodidae) in an American tegumentary leishmaniasis transmission area in northern Espírito Santo State, Brazil. Cad Saude Publica. 2008; 24: 2969-2978.

38. Souza CF, Quaresma PF, Andrade Filho JD, Bevilacqua PD. Phlebotomine fauna in the urban area of Timóteo, State of Minas Gerais, Brazil. Acta Trop. 2014; 134: 72-79.

39. Saraiva L, Andrade Filho JD, Silva SO, Andrade ASR, Melo MN. The molecular detection of different *Leishmania* species within sand flies from a cutaneous and visceral leishmaniasis sympatric area in Southeastern Brazil. Mem Inst Oswaldo Cruz.2010; 105: 1033-1039.

40. Margonari C, Soares RP, Andrade-Filho JD, Xavier DC, Saraiva L, Fonseca AL, et al. Phlebotomine Sand Flies (Diptera: Psychodidae) and *Leishmania* Infection in Gafanhoto Park, Divinópolis, Brazil. J Med Entomol. 2010; 47: 1212-1219.

41. Macedo-Silva VP, Martins DRA, De Queiroz PVS, Pinheiro MPG, Freire CCM, Queiroz JW, et al. Feeding Preferences of *Lutzomyia longipalpis* (Diptera: Psychodidae), the Sand Fly Vector for *Leishmania infantum* (Kinetoplastida: Trypanosomatidae). J Med Entomol. 2014; 51: 237-244.

42. Salomón OD, Feliciangeli MD, Quintana MG, Afonso MM dos S, Rangel EF. *Lutzomyia longipalpis* urbanisation and control. Mem Inst Oswaldo Cruz. 2015; 110: 831-846.

43. Gomes AC, Barata JMS, Rocha e Silva EO, Galati EAB. Aspectos ecológicos da leishmaniose tegumentar americana. 6. Fauna flebotomínea antropófila de matas residuais situadas na região centro-nordeste do Estado de são paulo, brasil. Rev Inst Med Trop São Paulo. 1989; 31: 32-39.

44. Moschin JC, Ovallos FG, Sei IA, Galati EAB, Moschin JC, Ovallos FG, et al. Ecological aspects of phlebotomine fauna (Diptera, Psychodidae) of Serra da Cantareira, Greater São Paulo Metropolitan region, state of São Paulo, Brazil. Rev Bras Epidemiol. 2013; 16: 190-201.

45. Carvalho MR, Valença HF, Silva FJ, Pita-Pereira D, Araújo Pereira T, Britto C, et al. Natural *Leishmania infantum* infection in *Migonemyia migonei* (França, 1920) (Diptera: Psychodidae: Phlebotominae) the putative vector of visceral leishmaniasis in Pernambuco State, Brazil. Acta Trop. 2010; 116: 108-110.

46. Carvalho GML, Andrade Filho JD, Falcão AL, Lima ACVMR, Gontijo CMF. Naturally Infected *Lutzomyia* Sand Flies in a Leishmania- Endemic Area of Brazil. Vector-Borne Zoonotic Dis. 2008; 8: 407-414.

47. Saraiva L, Carvalho GML, Quaresma PF, Rocha Lima ACVM, Falcão AL, Andrade-Filho JD. Natural infection of *Nyssomyia neivai* (Pinto, 1926) and *Evandromyia sallesi* (Galvão & Coutinho, 1939) (Diptera: Psychodidae) by *Leishmania infantum chagasi* (Cunha & Chagas, 1937) in Minas Gerais, Brazil. J Med Entomol. 2009; 46: 1159-1163.

48. Lana RS, Michalsky EM, Fortes-Dias CL, França-Silva JC, Lara-Silva FO, Lima ACVMR, Avelar DM, Martins JCD, Dias ES. Phlebotomine Sand Fly Fauna and Leishmania Infection in the Vicinity of the Serra do Cipó National Park, a Natural Brazilian Heritage Site. BioMed Res Int. 2015; 385493

49. Silva OS, Grunewald J. Contribution to the sand fly fauna (Diptera: Phlebotominae) of Rio Grande do Sul, Brazil and *Leishmania* (*Viannia*) infections. Mem Inst Oswaldo Cruz. 1999; 94: 579-582.

50. Rocha LS, Santos CB, Falqueto A, Grimaldi G, Cupolilo E. Molecular biological identification of monoxenous trypanosomatids and *Leishmania* from

antropophilic sand flies (Diptera: Psychodidae) in Southeast Brazi. Parasilto Res. 2010; 107: 465-468.

51. Rêgo FD, Souza GD, Dornelles LFP, Andrade Filho JD. Ecology and Molecular Detection of *Leishmania infantum* Nicolle, 1908 (Kinetoplastida: Trypanosomatida) in Wild-Caught Sand Flies (Psychodidae: Phlebotominae) Collected in Porto Alegre, Rio Grande do Sul: A New Focus of Visceral Leishmaniasis in Brazil. J Med Entomol 2019; 56: 519–525.

52. Carvalho, GML, Rêgo FD, Tanure A, Silva ACP, Dias TA, Paz GF, et al. Blood Meal Identification in Field-Captured Sand flies: cytochrome *b* PCR followed by DNA sequencing technique. J Med Entomol. 2017; 54: 1049-1054

53. Loiola CF, Silva DA, Galati EAB. Phlebotomine fauna (Diptera: Psychodidae) and species abundance in an endemic area of American cutaneous leishmaniasis in southeastern Minas Gerais, Brazil. Mem Inst Oswaldo Cruz. 2007; 102: 581-585.

54. Colla-Jacques FE, Casanova C, Prado AP. Study of sand fly fauna in an endemic area of American cutaneous leishmaniasis and canine visceral leishmaniasis in the municipality of Espírito Santo do Pinhal, São Paulo, Brazil. Mem Inst Oswaldo Cruz. 2010; 105: 208-215.

55. Fonteles RS, Pereira Filho AA, Moraes JL, P , Kuppinger O, and Rebêlo JMM. Experimental infection of *Lutzomyia (Nyssomyia) whitmani* (Diptera: Psychodidae: Phlebotominae) with *Leishmania* (*Viannia*) *braziliensis* and *Leishmania* (*L.*) *amazonensis*, etiological agents of American Tegumentary Leishmaniasis. J Med Entomol. 2016; 53: 206-209.

56. Quaresma, P. F., G. M. L. Carvalho, M. C. N. F. Ramos, and J. D. Andrade Filho. 2012. Natural *Leishmania* sp. reservoirs and phlebotomine sandfly food

source identification in Ibitipoca State Park, Minas Gerais, Brazil. Mem Inst Oswaldo Cruz. 107: 480-485.

57. Tonelli GB, Tanure A, Rêgo FD, Carvalho GML, Simões TC, Andrade Filho JD. Aspects of the ecology of phlebotomine sand flies (Diptera: Psychodidae) in the Private Natural Heritage Reserve Sanctuary Caraça. PLoS ONE 2017; 12: e0178628.

58. Lara-Silva FO, Michalskya, Fortes-Dias CL, Fiuza VOL, Pessanha JEM, Regina-Silva S. Epidemiological aspects of vector, parasite, and domestic reservoir in areas of recent transmission and no reported human cases of visceral.

59. Maruyama SR, Santana AKM, Takamiya NT, Takahashi TY, Rogerio LA, Oliveira CAB, et al. Non-*Leishmania* parasite in fatal visceral leishmaniasis–like disease, Brazil. Emerg Infect Dis. 2019;25: 2088–2092.

60. Ghobakhloo N, Motazedian MH, Naderi S, Sepideh E. Isolation of *Crithidia* spp. from lesions of immunocompetent patients with suspected cutaneous leishmaniasis in Iran. Trop Med Int Health. 2019; 24: 116–126.

61. Rêgo FD, Rugani JMN, Shimabukuro PHF, Tonelli GB, Quaresma PF, Gontijo CMF. Molecular detection of *Leishmania* in Phlebotomine sand flies (Diptera: Psychodidae) from a cutaneous leishmaniasis focus at Xakriabá Indigenous Reserve, Brazil. PLoS One; 2015. 10: e0122038.

Supporting Information Legends

S1License for the collection of sandflies granted by the Brazilian environmental agency (SISBIO)

S2 The copyright permission for figure 1.

S3 Revision English by native speaker

Table 1. Sand flies collected according to month at the Casa Branca, Brumadinho, Minas Gerais, from May 2013 to July 2014.

Species	Months of collection																		
	May/13		Jul/14		Sep/13		Nov/13		Jan/14		Mar/14		May/14		Jul/14		Total		
	♂	♀	♂	♀	♂	♀	♂	♀	♂	♀	♂	♀	♂	♀	♂	♀	♂	♀	Total (%)
Brumptomyia sp.	0	0	0	1	4	4	0	0	0	0	0	2	0	0	0	0	4	7	11 (0.1)
Cortelezzii complex	0	7	0	2	0	12	0	5	0	1	0	1	0	9	0	1	0	38	38 (0.2)
Evandromyia cortelezzii	1	0	2	0	3	0	1	0	2	0	1	0	0	0	0	0	10	0	10 (0.1)
Evandromyia edwarsi	0	0	0	0	0	0	0	0	0	0	0	0	0	1	0	0	0	1	1 (0.0)
Evandromyia evandroi	0	0	0	0	0	0	0	0	1	0	0	0	0	0	0	0	1	0	1 (0.0)
Evandromyia lenti	1	0	0	1	2	2	10	5	39	29	0	2	2	3	0	1	54	43	97 (0.6)
Evandromyia sallesi	1	0	0	0	2	0	0	0	0	0	0	0	1	0	1	0	5	0	5 (0.0)
Evandromyia teratodes	0	0	0	0	0	0	0	0	0	0	0	0	0	1	0	0	0	1	1 (0.0)
Evandromyia termitophila	0	1	0	1	0	1	0	5	0	0	0	0	0	3	0	0	0	11	11 (0.1)
Evandromyia tupynambai	0	1	0	0	3	0	0	2	0	2	0	0	1	10	0	0	4	15	19 (0.1)
Lutzomyia amarali	0	0	0	0	0	0	0	0	0	0	0	2	0	1	0	0	0	3	3 (0.0)
Lutzomyia ischyracantha	0	0	0	0	1	0	0	0	0	0	0	0	0	0	0	0	1	0	1 (0.0)
Lutzomyia longipalpis	528	127	57	5	430	112	181	41	63	22	214	62	547	104	55	3	2,075	476	2551 (15.2)
Migonemyia migonei	219	90	72	12	175	53	156	49	104	17	73	24	373	62	41	13	1213	320	1533 (9.1)
Nyssomyia intermedia	0	0	1	2	1	0	0	0	0	0	0	0	0	0	0	0	2	2	4 (0.0)
Nyssomyia whitmani	3,449	2,535	79	263	793	680	547	343	625	324	101	72	974	959	77	62	6,645	5,238	11,883 (70.9)
Pintomyia bianchigalatiae	0	13	0	0	0	10	1	2	0	3	0	17	0	30	0	0	1	75	76 (0.5)
Pintomyia fischeri	7	13	6	2	7	11	34	15	27	34	14	18	11	49	5	4	111	146	257 (1.5)
Pintomyia mamedei	0	1	0	0	0	0	0	0	0	0	0	0	0	0	0	0	0	1	1 (0.0)
Pintomyia misionensis	0	0	0	3	0	0	0	0	0	1	0	0	0	4	0	1	0	9	9 (0.1)
Pintomyia monticola	1	4	0	2	0	1	2	0	0	1	0	6	2	21	1	0	6	35	41 (0.2)
Pintomyia pessoai	1	1	0	0	1	18	0	19	3	0	1	0	1	3	0	0	7	41	48 (0.3)
Psathyromyia pascalei	0	0	0	0	1	0	0	0	0	1	0	0	1	0	1	0	3	1	4 (0.0)
Psathyromyia sp.	0	0	0	1	0	0	0	0	0	0	0	0	0	0	0	0	0	1	1 (0.0)
Psychodopygus lloydi	1	33	0	4	0	0	6	3	4	47	0	0	6	38	0	0	17	125	142 (0.9)
**Total**	4209	2845	217	299	1423	908	938	489	868	482	404	206	1919	1298	181	85	10159	6612	16771 (100)
	**7,054**		**516**		**2,331**		**1427**		**1,350**		**610**		**3,217**		**266**		**16,771**		

Table 2. Sand fly organized by species that were analyzed individually and in pools collected in Casa Branca, Brumadinho, Minas Gerais, from May 2013 to May 2014.

Species	Sand flies collected	number of analyzed pools	specimens analyzed individually
Brumptomyia sp*	4	1	0
Cortelezzii complex	25	5	3
Evandromyia edwardsi	1	0	1
Evandromyia lenti	31	4	3
Evandromyia teratodes	1	0	1
Evandromyia termitophila	6	1	4
Evandromyia tupynambai	13	2	1
Lutzomyia amarali	1	0	1
Lutzomyia longipalpis	248	19	0
Migonemyia migonei	144	14	0
Nyssomyia whitmani	4128	220	0
Pintomyia bianchigalatiae	47	8	2
Pintomyia fischeri	80	13	0
Pintomyia misionensis	4	0	4
Pintomyia monticola	27	3	3

Pintomyia pessoai	16	3	1
Pintomyia sp.*	21	0	21
Psathyromyia pascalei	1	0	1
Psychodopygus lloydi	115	18	1
**Total**	**4913**	**311**	**47**

* sand flies damaged

Table 3. Results of DNA detection and identification of species of Trypanosomatidae according to species of sand fly, number of pool and collection date in Casa Branca, Brumadinho from May 2013 to May 2014.

Accession numbers	% similarity	Seq ID Blast	Host	Collection date
MN638721	97,31	*Crithidia* sp.	*Nyssomyia whitmani*	may-2013
MN638722	95,38	*Herpetomonas* sp.	Complexo cortelezzii	may-2013
MN638723	90,21	*Herpetomonas* sp.	*Nyssomyia whitmani*	may-2013
MN638724	99,51	*Leishmania* sp.	*Nyssomyia whitmani*	may-2013
MN638725	99,6	*Leishmania* sp.	*Nyssomyia whitmani*	may-2013
MN638726	100	*Leishmania amazonensis*	*Nyssomyia whitmani*	may-2013
MN638727	100	*Leishmania amazonensis*	*Nyssomyia whitmani*	may-2013
MN638728	96,5	*Crithidia* sp.	*Psychodopygus lloydii*	may-2014
MN638729	97	*Herpetomonas* sp.	*Nyssomyia whitmani*	may-2013
MN638730	99,32	*Leishmania braziliensis*	*Psychodopygus lloydii*	may-2014
MN638731	99,66	*Leishmania* sp.	*Nyssomyia whitmani*	may-2013
MN638732	99,32	*Leishmania* sp.	*Nyssomyia whitmani*	may-2013
MN638733	80,2	*Leishmania* sp.	*Lutzomyia longipalpis*	may-2013
MN638734	98,01	*Leishmania amazonensis*	*Nyssomyia whitmani*	sep-2013
MN638735	99,04	*Leishmania* sp.	*Nyssomyia whitmani*	may-2013
MN638736	98,45	*Leishmania* sp.	*Nyssomyia whitmani*	may-2013
MN638737	98,72	*Leishmania* sp.	*Nyssomyia whitmani*	may-2013
MN638738	99,39	*Leishmania amazonensis*	*Nyssomyia whitmani*	may-2013

Legend: Seq ID Blast = hit provided by Blast search.

[1] Trypanosomatid sequence obtained from strain A9 from the Coleção de Protozoários (FIOCRUZ/COLPROT).

Supporting Information

Fig. 1 - Location of Casa Branca district on the map of the state of Minas Gerais, Brazil.

Fig. 2 - Standardized index of species abundance of species collected at Casa Branca, Brumadinho, Minas Gerais between May 2013 and July 2014.

Fig. 3 - The relationship between seasonal variation in the number of individuals of species of sand flies collected and the climate variables at Casa Branca, Brumadinho, Minas Gerais in May 2013 to July 2014.

PONE-D-20-05836
Diversity of phlebotomine sand flies and molecular detection of trypanosomatids species in Brumadinho, Minas Gerais, Brazil
PLOS ONE

Dear Vyacheslav Yurchenko,

Below are our answers to requests from the Editor and the PLOS ONE technical team.

Thanks

Additional Editor Comments (if provided):

Reading the manuscript of Tanure at al I noticed the huge unbalance in the literature citations. Authors (almost exclusively) cite Brazilian papers effectively ignoring the huge amount of work on this topic, which has been done in other parts of the world. I invite authors to correct this discrepancy and submit a revised version for peer review.
V, Yurchenko

The Editor is right, there were many references in Portuguese that could be replaced by others, thanks for the suggestion.

Journal Requirements:

When submitting your revision, we need you to address these additional requirements:

1. Please ensure that your manuscript meets PLOS ONE's style requirements, including those for file naming. The PLOS ONE style templates can be found at http://www.plosone.org/attachments/PLOSOne_formatting_sample_main_body.pdf and http://www.plosone.org/attachments/PLOSOne_formatting_sample_title_authors_affiliations.pdf

We understand that our article and files are based on the PLOS ONE style.

2. One of the noted authors is a group or consortium [Grupo de Estudos em Leishmanioses]. In addition to naming the author group, please list the individual authors and affiliations within this group in the acknowledgments section of your manuscript. Please also indicate clearly a lead author for this group along with a contact email address.

Grupo de Estudos em Leishmanioses is the author's affiliation, a name similar to "laboratory". All authors belong to the leishmaniasis study group (Grupo de Estudos em Leishmanioses)

3. Please amend your list of authors on the manuscript to ensure that each author is linked to an affiliation. Authors' affiliations should reflect the institution where the work was done (if authors moved subsequently, you can also list the new affiliation stating "current affiliation:…." as necessary).

All authors are listed and with their affiliation

4. Please ensure that you refer to Figure 2 in your text as, if accepted, production will need this reference to link the reader to the figure.

We have included the reference to figure 2 in the text, line 214

5. We note that Figure 1 in your submission contain map images which may be copyrighted. All PLOS content is published under the Creative Commons Attribution License (CC BY 4.0), which means that the manuscript, images, and Supporting Information files will be freely available online, and any third party is permitted to access, download, copy, distribute, and use these materials in any way, even commercially, with proper attribution. For these reasons, we cannot publish previously copyrighted maps or satellite images created using proprietary data, such as Google software (Google Maps, Street View, and Earth). For more information, see our copyright guidelines: http://journals.plos.org/plosone/s/licenses-and-copyright.

We require you to either (1) present written permission from the copyright holder to publish these figures specifically under the CC BY 4.0 license, or (2) remove the figures from your submission:

1. You may seek permission from the original copyright holder of Figure 1 to publish the content specifically under the CC BY 4.0 license.

We recommend that you contact the original copyright holder with the Content Permission Form (http://journals.plos.org/plosone/s/file?id=7c09/content-permission-form.pdf) and the following text:

"I request permission for the open-access journal PLOS ONE to publish XXX under the Creative Commons Attribution License (CCAL) CC BY 4.0 (http://creativecommons.org/licenses/by/4.0/). Please be aware that this license allows unrestricted use and distribution, even commercially, by third parties. Please reply and provide explicit written permission to publish XXX under a CC BY license and complete the attached form."

Please upload the completed Content Permission Form or other proof of granted permissions as an "Other" file with your submission.

In the figure caption of the copyrighted figure, please include the following text: "Reprinted from [ref] under a CC BY license, with permission from [name of publisher], original copyright [original copyright year]."

2. If you are unable to obtain permission from the original copyright holder to publish these figures under the CC BY 4.0 license or if the copyright holder's requirements are incompatible with the CC BY 4.0 license, please either i) remove the figure or ii) supply a replacement figure that complies with the CC BY 4.0 license. Please check copyright information on all replacement figures and update the figure caption with source information. If applicable, please specify in the figure caption text when a figure is similar but not identical to the original image and is therefore for illustrative purposes only.

We will provide copyright permission for figure 1 and include it in the submission system

Thank you!!!!

José Dilermando Andrade Filho
