# Supplementary material for: Diversity of phlebotomine sand flies and molecular detection of trypanosomatids in Brumadinho, Minas Gerais, Brazil
Source: PLoS One. 2020 Jun 24;15(6):e0234445. doi: 10.1371/journal.pone.0234445 (PMC7314019; doi:10.1371/journal.pone.0234445)
Supplement: Attachment — Submitted filename: Rebutal.doc [file pone.0234445.s001.doc]

Dear editor,

Below my considerations

It has come to our attention that the manuscript seems to be a resubmission of the manuscript “PONE-D-16-42847R1 ”, which was previously considered by the journal.

It is a requirement by the journal that any manuscript resubmitted after a previous rejection is accompanied by a rebuttal letter outlining the response to the original reviewers’ comments, so that the editors can establish whether the original concerns have been addressed. Can you therefore please provide us with a point-by-point response to the comments provided by the original reviewers, outlining the revisions carried out since the original submission.

**The corresponding author did not inform about previous submission because he believes that the articles are quite different. Previously, a version based only on the diversity of sand flies in the studied area was presented. In this version, data on diversity were reduced and included on the detection of *Leishmania* DNA in females of sand flies found in the collections.**

**Follow my responses to the referees' comments.**

Reviewer #1: The authors thought the study would be unfeasible if the sand flies were collected biweekly or monthly because the sand flies collected in the first month were too many. The excuse is not acceptable. Most adult sand flies can only survive for 2 to 3 weeks. If collected bimonthly, the species of sand fly surviving in the interval may not be collected, and lead to the wrong conclusions which these species of sand fly can not survive in the area . The researchers could not sacrifice the preciseness and scientific principles because of the excessive workload. The problem of excessive workload can be solved by adding staff. The authors listed several articles in which the interval time for collection of sand flies was long to support their own views, but overlong interval time for collection was bound to loss some important information, and affect preciseness of the study. In fact, most of the researchers, including corresponding author in this manuscript, collected sand flies biweekly or monthly in most of their study. So, I think this manuscript is not precise enough to meet the criteria published in PLOS One.

**Reviewer # 1 made an absurd, pointless comment and the editor at the time was right. We often have to adapt the methodology to the particularities of each area. I send the same answer that I sent to the same when the evaluation**

**The time interval between the collections, which was every two months, was decided based on the large number of sand flies collected in the first month (approximately 7,000 sand flies). It would be unfeasible to prepare, mount and identify all the sand flies in the limited time we had to present results, the article in question is the result of the master's dissertation of the first author. Also, we believe that the total amount of insects collected (16,771) in the present work are providing meaningful, relevant information about the studied fauna, regardless if collections were made biweekly or monthly, as the reviewer suggested.**

**Moreover, several studies are carried out with bimonthly collections or even one in each season of the year (see Alves et al. 2012; Carvalho et al. 2012; Machado et al. 2012; Rêgo et al. 2014). Our point of view is that the collections were systematic, i.e. they were performed every two months.**

Reviewer #3: Authors in this manuscript studied the diversity of Phlebotomine sand flies, the vector for Leishmania parasites, in the district of Casa Branca in the state of Minas Gerais, Brazil. Number of flies trapped and tested are reasonable. However, the presence of the parasite or parasite DNA were not inve3stigated. Furthermore, different species of sand flies were identified by only morphological characteristics. Overall studies provide a good sense of diversity and abundance of different species of sand flies in the above mentioned district, however, its link to the spreading of Leishmaniasis is rather limited.

**The Leishmania DNA detection part has been included in this version of the article.**

Thank you

José Dilermando Andrade Filho
