# Supplementary material for: Diversity of phlebotomine sand flies and molecular detection of trypanosomatids in Brumadinho, Minas Gerais, Brazil
Source: PLoS One. 2020 Jun 24;15(6):e0234445. doi: 10.1371/journal.pone.0234445 (PMC7314019; doi:10.1371/journal.pone.0234445)
Supplement: Attachment — Submitted filename: Response to Reviewers.doc [file pone.0234445.s002.doc]

PONE-D-20-05836
Diversity of phlebotomine sand flies and molecular detection of trypanosomatids species in Brumadinho, Minas Gerais, Brazil
PLOS ONE


**Dear Vyacheslav Yurchenko,**

**Below are our answers to requests from the Editor and the PLOS ONE technical team.**

**Thanks**

Additional Editor Comments (if provided):

Reading the manuscript of Tanure at al I noticed the huge unbalance in the literature citations. Authors (almost exclusively) cite Brazilian papers effectively ignoring the huge amount of work on this topic, which has been done in other parts of the world. I invite authors to correct this discrepancy and submit a revised version for peer review. V, Yurchenko

**The Editor is right, there were many references in Portuguese that could be replaced by others, thanks for the suggestion.**

Journal Requirements:

When submitting your revision, we need you to address these additional requirements:

1. Please ensure that your manuscript meets PLOS ONE's style requirements, including those for file naming. The PLOS ONE style templates can be found at http://www.plosone.org/attachments/PLOSOne_formatting_sample_main_body.pdf and http://www.plosone.org/attachments/PLOSOne_formatting_sample_title_authors_affiliations.pdf

**We understand that our article and files are based on the PLOS ONE style.**

2. One of the noted authors is a group or consortium [Grupo de Estudos em Leishmanioses]. In addition to naming the author group, please list the individual authors and affiliations within this group in the acknowledgments section of your manuscript. Please also indicate clearly a lead author for this group along with a contact email address.

**Grupo de Estudos em Leishmanioses is the author's affiliation, a name similar to "laboratory". All authors belong to the leishmaniasis study group (Grupo de Estudos em Leishmanioses)**

3. Please amend your list of authors on the manuscript to ensure that each author is linked to an affiliation. Authors’ affiliations should reflect the institution where the work was done (if authors moved subsequently, you can also list the new affiliation stating “current affiliation:….” as necessary).

**All authors are listed and with their affiliation**

4. Please ensure that you refer to Figure 2 in your text as, if accepted, production will need this reference to link the reader to the figure.

**We have included the reference to figure 2 in the text, line 214**

5. We note that Figure 1 in your submission contain map images which may be copyrighted. All PLOS content is published under the Creative Commons Attribution License (CC BY 4.0), which means that the manuscript, images, and Supporting Information files will be freely available online, and any third party is permitted to access, download, copy, distribute, and use these materials in any way, even commercially, with proper attribution. For these reasons, we cannot publish previously copyrighted maps or satellite images created using proprietary data, such as Google software (Google Maps, Street View, and Earth). For more information, see our copyright guidelines: <http://journals.plos.org/plosone/s/licenses-and-copyright>.

We require you to either (1) present written permission from the copyright holder to publish these figures specifically under the CC BY 4.0 license, or (2) remove the figures from your submission:

1.    You may seek permission from the original copyright holder of Figure 1 to publish the content specifically under the CC BY 4.0 license.

We recommend that you contact the original copyright holder with the Content Permission Form (http://journals.plos.org/plosone/s/file?id=7c09/content-permission-form.pdf) and the following text:

“I request permission for the open-access journal PLOS ONE to publish XXX under the Creative Commons Attribution License (CCAL) CC BY 4.0 (http://creativecommons.org/licenses/by/4.0/). Please be aware that this license allows unrestricted use and distribution, even commercially, by third parties. Please reply and provide explicit written permission to publish XXX under a CC BY license and complete the attached form.”

 Please upload the completed Content Permission Form or other proof of granted permissions as an "Other" file with your submission.

In the figure caption of the copyrighted figure, please include the following text: “Reprinted from [ref] under a CC BY license, with permission from [name of publisher], original copyright [original copyright year].”

2.    If you are unable to obtain permission from the original copyright holder to publish these figures under the CC BY 4.0 license or if the copyright holder’s requirements are incompatible with the CC BY 4.0 license, please either i) remove the figure or ii) supply a replacement figure that complies with the CC BY 4.0 license. Please check copyright information on all replacement figures and update the figure caption with source information. If applicable, please specify in the figure caption text when a figure is similar but not identical to the original image and is therefore for illustrative purposes only.

**We will provide copyright permission for figure 1 and include it in the submission system**

Thank you!!!!

José Dilermando Andrade Filho
