# Supplementary material for: Diversity of phlebotomine sand flies and molecular detection of trypanosomatids in Brumadinho, Minas Gerais, Brazil
Source: PLoS One. 2020 Jun 24;15(6):e0234445. doi: 10.1371/journal.pone.0234445 (PMC7314019; doi:10.1371/journal.pone.0234445)
Supplement: Attachment — Submitted filename: Response to Editor.doc [file pone.0234445.s004.doc]

René Rachou Institute-FIOCRUZ/MG

Avenida Augusto de Lima, n° 1715, Barro Preto

Belo Horizonte/MG/Brazil

CEP 30190-002

12^th^ May 2020

Manuscript ID: PONE-D-20-05836R1

Dear Dr. Yurchenko,

We would like to thank for your thoughtful review of the manuscript. You raised important issues and your inputs were well-considered and gratefully implemented. We agree with almost all your comments and we have revised our manuscript accordingly.

We have crafted a revised version of the paper stating the hypothesis and the implications of our work more clearly than before, including the suggestions. We are confident that the new version of the manuscript is greatly improved. We respond below in details to each of your comments. If we have slightly disagreed with something, we stated why. We hope that you will find our responses to your comments satisfactory, and we are willing to finish the revised version of the manuscript.

Please, find below your comments repeated in italics and our responses inserted after each comment. To facilitate the revision process, in some instances we refer to the plain text file indicating the page and the line (page-line).

Looking forward to hearing from you soon.

Sincerely,

José Dilermando Andrade Filho

***Additional Editor Comments (if provided):*** ***Please address all the concerns raised by 3 reviewers. In addition, I request: 1) to extend the introduction on trypanosomatids and discuss the following publications: Maslov et al, 2019 (Parasitology) and Lukes et al, 2018 (Trends Prasitol). 2) to spell all species names in Italic; iii) fix the references; iv) if you cite ref 60, please cite an accompanying paper- Kostygov et al, 2019 (Trop Med Int Health).***

**Authors’ comment:** We accept all your suggestions. The introduction has been improved with the mentioned papers; all species names and the references have been revised as well. Information about the paper Kostygov et al. 2019 was also added in the manuscript (now lines 58-59).

***Reviewer #1: This is a nice study of a sand fly fauna (in the Casa Branca district of Brumadinho municipality) demonstrating the high phlebotomine diversity and the presence of DNA of two Leishmania species as well as several (3-4) non-Leishmania (monoxenous) parasites. However, because the design of the study (bimonthly = visiting of the locality once per two months) is not sufficient for any “ecological” analysis, I would suggest deleting all parts connected with “seasonal” variation in sand fly abundance and climate (and other) data. The number of collections is really not sufficient for any similar analysis; but even without these unreliable and therefore misleading results, the study is fully reliable to be published. However, there are several less important unclarities to must be solved.***

**Authors’ comment:** We totally agree with your comment. All data about the relationship between seasonal variation and sand fly abundance have been removed.

***4 trypanosomatid species or trypanosomatids***

**Authors’ comment:** The title has been changed (now line 3).

***32 HP – meaning***

**Authors’ comment:** The sentence has been changed (now line 20-22).

***33 better trap/nights (to demonstrate the effort)***

**Authors’ comment:** The sentence has been changed (now lines 20-22).

***39-40 how many individuals and how many in pools (BTW, pools of 1 specimen is not a pool !!!) (******Of a total of 4,913 females, 47 were individually tested and 4,866 grouped in 311 pools).***

**Authors’ comment:** The sentence has been changed (now lines 20-24).

***42 instead of Leishmania sp. use … in seven pools of Ny. whitmani and one pool of Lu. longipalpis the leishmania species was not determined (to the low quality of the sequences) … or something like this, otherwise, it seems that some “new/unknown” leishmania species was detected 43.***

**Authors’ comment:** The sentence has been changed (now lines 28-30).

***44-45 same type of result presentation as in the case of Leishmania spp. – it means: how many pools/individuals and in which sand fly species.***

**Authors’ comment:** The sentence has been changed (now lines 27-33).

***45 The presence of DNA is not proving of vector capacity! (…*** ***may be involved in the transmission of Le. amazonensis … etc. is much more accurate)***

**Authors’ comment:** The sentence has been changed (now line 34-36).

***55-57 REFs 1-3 are more-or-less relevant, but there are several current reviews, e.g., Akhoundi et al. 2016 (PLoS Neglected Tropical Diseases 10(3): e0004349.), etc… that would fit here.***

**Authors’ comment:** The references have been changed (now lines 43-46).

***59 REF 4 – ok, but add also e.g., Rangel, Shaw (2018) Brazilian Sand Flies … (10.1007/978-3-319-75544-1) etc.***

**Authors’ comment:** The reference has been added (now line 49).

***71-72 The genus Trypanosoma is missing! Sand flies are possible vectors of several trypanosome species – add a relevant reference(s) (the sentence “Several authors have reported the presence of non-Leishmania trypanosomatids DNA in sand flies by means of molecular techniques [12, 13, 14, 15]” does not cover this issue clear enough)***

**Authors’ comment:** The sentence has been changed and references covering this issue have been added (now lines 50-70).

***73 REFs 10 and 11 – OK, but add some references on Leptomonas (e.g. Kraeva et al. 2015 (PLoS Pathog 11(8):e1005127)); and even more references would be appropriate here***

**Authors’ comment:** The reference was added (now line 70).

***71-73 differentiate between monoxenous and dixenous genera***

**Authors’ comment:** This information has been added (now lines 50-53).

***77-81 The statement is controversial in some aspects – first of all, the situation with the genus “Endotrypanum” is rather complicated (see doi: 10.14411/fp.2017.020). And in addition to this, the sentence, which is articulated in this way and is given in the previous contexts, makes an impression on readers that the authors refer primarily to single-host (monoxenous) genera (such as Crithidia, Leptomonas, etc.).***

**Authors’ comment:** The paragraph has been deeply revised. Now, we believe that the background context that we provided make clear our goal (now lines 50-70).

***124 CDC light traps (model HP) – I am sure that there is no official model HP within the CDC traps – it is an only modification based on the CDC trap design – which company produces these traps or is it just some home-made version?***

**Authors’ comment:** We agree with your comment. The automatic light trap (model HP) is an improved model of suction light trap for the capture of small insects, similar to CDC light traps. However, now in the manuscript text, we referred to those traps as “automatic light traps (model HP)” and not CDC light traps (model HP). This information could be check in the lines 99-101.

***125-6 not clear – it means that the traps run three nights (and two days)?
Sand flies were still alive (after three days???), How many sand flies survive till laboratory (approximately in % ???)***

**Authors’ comment:** The sentence has been improved (now lines 99-102). Many sand flies still alive after three days, but we could not provide the percentage since it was not evaluated. Furthermore, to carry out molecular techniques, living sand flies is unnecessary.

***137-8 when the morphological identification was made? The key is from the end of 2018, the collection and slide preparation was made much earlier (as was mentioned in the previous response to reviewers)!!!***

**Authors’ comment:** The reference Galati (2018) is a book chapter including updates of Galati (2003). In the previous review, the changing of the oldest reference for a newest one was suggested, especially because it is a Portuguese version, so we did it. Even so, we rewrite the sentence highlighting this information (now lines 110-111).

***158 … of trypanosomatids (not Leishmania)***

**Authors’ comment:** The sentence was been changed (now line 116).

***247 – the prevalence is right, but anyway it should be explained that the authors predict that*** ***only one specimen per pool was infected… (it corresponds with the low “pool” prevalence)***

**Authors’ comment:** We totally agree with your comment. However, in the new version we presented the prevalence per positive pool (now lines 183-198).

***253/255 – why “it was not possible get to species level”? - due to the low quality of the sequences ??? or the sequences represent some new leishmania species ??? It must be explained!***

**Authors’ comment:** All sequences without species level identification presented low quality, containing high number of ambiguous sites. We stated this information in the lines 185-188.

***258-9 “the presence of bands of inconsistent size (400-500 bp) with fragments for Leishmania (~350 bp)” – not clear. Does it mean that the double bands were detected = mixed infection of leishmania (350bp) + some monoxenous trypanosomatids ??? And if yes, it means that monoxenous trypanosomatids occur only in pools positive for leishmania parasites???? It is really very unusual and must be discussed!***

**Authors’ comment:** We observed that the non-*Leishmania* parasites shown amplicons of 400-500 pb, distinct to predicted for *Leishmania* parasites (300-350pb). And this fact, led us to investigate what was amplified. There was no double-bands/mixed infections. However, the sentence has been changed (192-195).

***260 - … three trypanosomatids … - must be clearly distinguished even in Tab. 3 – e.g., Herpetomonas sp. 1, Herpetomonas sp. 2 …***

**Authors’ comment:** The sentence has been changed (now lines 193-195).

***263/352 – Herpetomonas sp. means unidentified ONE species of the genus Herpetomonas, but it seems that two different Herpetomonas species were detected – thus Herpetomonas spp.!!!! Or better cortezzii – H. sp. 1, Ny.w. – H. sp. 2, to make it clear. The same for Crithidia – it is not clear if Crithidia parasites from Ps.l. and Ny.w. belong to one or two species – if the sequences represent two species, it would be better to use C. sp. 1 and C. sp. 2 to make it clear. You have to make it clear, especially if you are not present any phylogenetic tree etc.***

**Authors’ comment:** The sentence was been changed (now lines 192-198). As the sequences have aligned with “*Herpetomonas* sp.” and “*Crithidia* sp.” on Blast tool, we are not sure about the presence of more than one species within both genera of trypanosomatids. So, we decide to keep “sp.” referring at least one species of each genera.

***Table 3 – sort it according to “Host” or according to “trypanosomatid parasites!, not according Acc.Nos., it makes no sense.***

**Authors’ comment:** We accepted your suggestion. The table 3 has been changed according hosts.

***Seq ID Blast – the different unnamed Herpetomonas/Crithidia/Leishmania species (identified by BLAST) must be specified (by its voucher name/number etc., or even by their GB Acc.No.)!***

**Authors’ comment:** We are not sure about your inquiry, since all species identified by Blast tool have already been identified by their accession numbers, as presented in Table 3.

**Reviewer #2: I appreciate that authors improved the manuscript and add more international references. However, some of them were not chosen correctly. I suggest correct several references in the first half of the Introduction:**

**Authors’ comment:** We accept your suggestion. We add more international references when it was possible, especially in the first half of the introduction. However, for several aspects about sand fly fauna is desirable to use national references.

***Line 55: Ref. 2 (Alkan et al) is not an optimal reference. Would be better to include the famous review by Maroli et al. 2013, see the pdf.***

**Authors’ comment:** The reference has been added (now line 43-46).

***Line 57: Ref. 3 (Lestinova et al) is not suitable reference. Again, the review by Michele Maroli could be used.***

**Authors’ comment:** The sentence has been changed (now line 46).

***Line 59: Ref. 4. There is more recent review on this topic: chapter by Dvorak et al, published in Bruschi Fabrizio, Gradoni Luigi (eds.), The Leishmaniases: Old Neglected Tropical Diseases. Springer 2018.***

**Authors’ comment:** The reference has been added (now line 49).

***Lines 67-70: Please use some more recent reference. All this information is described in details by Jeffrey Shaw in the review by Dvorak et al.***

**Authors’ comment:** The reference has been added (now line 49).

***In Discussion, I suggest to improve discussion about Mg. migonei vector competence to L. infantum (Lines 292-294). The vectorial status of Mg. migonei was recently proven experimentally by Fitipaldi Veloso Guimaraes et al, 2016. Please, add this reference (or replace the existing one, no. 45).***

**Authors’ comment:** Discussion about *Mg. migonei* has been improved (now lines 230-238).

***Finally, I suggest shorten the “Short title” as it is too long now.***

**Authors’ comment:** The short title has been reviewed (now line 1).

***For more details and some other minor corrections of typos, see the pdf file with my comments.***

**Authors’ comment:** All suggestions marked in pdf file have been accepted.

***Reviewer #3: I gather that it is difficult to write a readable paper out of huge but relatively boring data, but the authors still shall try to do a better job. In general, the authors describe frequently and extensively things that can be found on Wikipedia (geography, how many people live in some district - the number is non-scientific because at the time of writing it was already incorrect), please remove all this, which unncessarily extends the manuscript and brings absolutely no novelty and has no relevance to the presented data (where is a correlation between the size of the district and phlebotomine sandflies?).***

**Authors’ comment:** We accepted your suggestion. We did a deeply revision in the manuscript file.

***I would like to ask the authors to better balance the Results and the Discussion, the first being too short and the latter too long.***

**Authors’ comment:** We accepted your suggestion. We did a deeply revision in the manuscript file.

***Discussing usefulness or not of PCR-RFLP and restriction enzymes is way outdated, this type of discussions were concluded 15 or rather 20 years ago and is irrelevant nowadays, when it is sequencing or nothing. At least as long as you want to publish in respected journals, such as PloS One. Hence, shorten this part of discussion by 90%. Again, sentences such as “DNA sequencing has generated good results when used for detection of 380 Leishmania in sand flies“ are funny and make no sense these days, sequencing when done properly always (!) generates good results, namely the letter AGCT.***

**Authors’ comment:** We accepted your suggestion. We did a deeply revision in the discussion section.

***Furthermore, the acknowledgements and technical details are extremely long and tedious, nobody says that gels were stained with EtBr anymore, this is a common knowledge - please avoid this South American stretching of the manuscripts, remove what is the capital etc. etc.***

**Authors’ comment:** We totally disagree with your comment. With all due respect, we do not understand how 2-3 lines of acknowledgments are quite boring. This is a personal section and we thank everyone who we would like. About gel electrophoresis, the most used staining for sure is EtBr however, it is not the only one. Alternative DNA dyes, such as SYBR Safe is also currently used. So, we decide to keep this information because it is not trivial, and it is not a "South American stretching" as you said. However, as we said in the last inquiries, we carefully revised the manuscript and we hope this version stated better our goal.

***Give more attention to the really interesting stuff regarding the parasites found. There is tons of references, which are overlaping with their info (+ IBGE reference – accessed in 2016? What is this?) This and some others are not standard type references and has to be deleted. Refs are in different fonts, formats, excessively long list describing all the time the same thing. Yet there is absolutely no discussion and literature about the potentially interesting findings of non-Leishmania trypanosomatids, which is a mistake that has to be rectified.***

**Authors’ comment:** The discussion section has been improved. Now, we discuss about the finding of non-*Leishmania* trypanosomatids (now lines 252-261).
