# Supplementary material for: Diversity of phlebotomine sand flies and molecular detection of trypanosomatids in Brumadinho, Minas Gerais, Brazil
Source: PLoS One. 2020 Jun 24;15(6):e0234445. doi: 10.1371/journal.pone.0234445 (PMC7314019; doi:10.1371/journal.pone.0234445)
Supplement: Attachment — Submitted filename: Response to Reviewers.doc [file pone.0234445.s005.doc]

René Rachou Institute-FIOCRUZ/MG

Avenida Augusto de Lima, n° 1715, Barro Preto

Belo Horizonte/MG/Brazil

CEP 30190-002

2^th^ May 2020

Manuscript ID: PONE-D-20-05836R1

Dear Dr. Yurchenko,

Dear Editor,

below is our response to the reviewers' comments.

We are available for clarification.

Thank you

Sincerely,

José Dilermando Andrade Filho

**Reviewer #1: I am more-or less satisfied with the current version, but I do not fully agree with one specific explanation.
Based on the sequences, which are already available in the GenBank database, it is clear that the sequences of monoxenous trypanosomatids differ from each other (within the three Herpetomonas spp. as well as between the two Crithidia spp.). Therefore, this information should be mentioned somewhere in the text, i.e., that the found Herpetomonas spp. and Crithidia spp. probably represent three and two, respectively, species, or at least different populations (or the Molecular Operational Taxonomic Unit; MOTU, could be used in this case), to make it clear that these sequences do not represent the same herpetomonas or Crithidia species. And, accordingly, Herpetomonas spp. and Crithidia spp. must be used in the text.
I would also recommend changing the sentence in the abstract
Instead of
“In seven pools of Ny. whitmani and in one pool of Lu. longipalpis the Leishmania species was not determined due to the low quality of the sequences.”
change like:
“In seven pools of Ny. whitmani and in one pool of Lu. longipalpis positive for leishmania DNA, the parasite species was not determined due to the low quality of the sequences.”**

We agree with your comment. It is possible that are more than one monoxenous trypanosomatids within our molecular detection. However, based on the small fragment (~400 pb) it is not of the sequence it is not wise to assume this fact. We hope in the future we are able to isolate these trypanosomatids and confirm their identity as species level. We include the phrase in results "These five non-Leishmania sequences found in our study, Herpetomonas spp. and Crithidia spp., are sufficiently different between them and possibly represent two and three, respectively, different MOTUs (Molecular Operational Taxonomic Units)." In the discussion we include “Possibly, more than one species within each genera were detected, however, due to the small length of the amplicon it was not possible to confirm it. On the other hand, the molecular detections presented here, may represented different populations (different MOTUs), reinforcing the importance of further studies on these trypanosomatids.”

We changed that phrase in the abstract “In seven pools of Ny. whitmani and in one pool of Lu. longipalpis positive for leishmania DNA, the parasite species was not determined due to the low quality of the sequences.”

**Reviewer #3: I find the way the authors responded acceptable and consider the paper much improved. I would still have some issues with the length of acknowledgements (theoretically you can have it as long as the discussion, there is no official limit, but it is simply not good), the way the gel is stained is irrelevant - it was stained somehow and that's all that matters, but I can live with the present version.**

We thank the reviewer for the comments
