# Supplementary material for: Diversity of phlebotomine sand flies and molecular detection of trypanosomatids in Brumadinho, Minas Gerais, Brazil
Source: PLoS One. 2020 Jun 24;15(6):e0234445. doi: 10.1371/journal.pone.0234445 (PMC7314019; doi:10.1371/journal.pone.0234445)
Supplement: Attachment — Submitted filename: Response to Reviewers.doc [file pone.0234445.s006.doc]

René Rachou Institute-FIOCRUZ/MG

Avenida Augusto de Lima, n° 1715, Barro Preto

Belo Horizonte/MG/Brazil

CEP 30190-002

22^th^ May 2020

Manuscript ID: PONE-D-20-05836R1

***Dear Dr. Yurchenko,***

***We have reviewed all of your requests.***

***All references have been fixed. Now, the generic and species names are in italic; we have also removed the capitalized words in some reference titles and diacritics have been reviewed.***

***We are confident that the new version of the manuscript is greatly improved.***

***Looking forward to hearing from you soon.***

**José Dilermando Andrade Filho**
